# Enhanced Healthspan in *Caenorhabditis elegans* Treated With Extracts From the Traditional Chinese Medicine Plants *Cuscuta chinensis* Lam. and *Eucommia ulmoides* Oliv.

**DOI:** 10.3389/fphar.2021.604435

**Published:** 2021-02-02

**Authors:** Shimaa M. A. Sayed, Karsten Siems, Christian Schmitz-Linneweber, Walter Luyten, Nadine Saul

**Affiliations:** ^1^ Molecular Genetics Group, Institute of Biology, Faculty of Life Sciences, Humboldt University of Berlin, Berlin, Germany; ^2^ Botany and Microbiology Department, Faculty of Science, New Valley University, El-Kharga, Egypt; ^3^ AnalytiCon Discovery GmbH, Potsdam, Germany; ^4^ Biology Department, KU Leuven, Leuven, Belgium

**Keywords:** C. *elegans*, *Cuscuta chinensis*, *Eucommia ulmoides*, healthspan, traditional Chinese medicine, aging

## Abstract

To uncover potential anti-aging capacities of Traditional Chinese Medicine (TCM), the nematode *Caenorhabditis elegans* was used to investigate the effects of *Eucommia ulmoides* and *Cuscuta chinensis* extracts, selected by screening seven TCM extracts, on different healthspan parameters. Nematodes exposed to *E. ulmoides* and *C. chinensis* extracts, starting at the young adult stage, exhibited prolonged lifespan and increased survival after heat stress as well as upon exposure to the pathogenic bacterium *Photorhabdus luminescens*, whereby the survival benefits were monitored after stress initiation at different adult stages. However, only *C. chinensis* had the ability to enhance physical fitness: the swimming behavior and the pharyngeal pumping rate of *C. elegans* were improved at day 7 and especially at day 12 of adulthood. Finally, monitoring the red fluorescence of aged worms revealed that only *C. chinensis* extracts caused suppression of intestinal autofluorescence, a known marker of aging. The results underline the different modes of action of the tested plants extracts. *E. ulmoides* improved specifically the physiological fitness by increasing the survival probability of *C. elegans* after stress, while *C. chinensis* seems to be an overall healthspan enhancer, reflected in the suppressed autofluorescence, with beneficial effects on physical as well as physiological fitness. The *C. chinensis* effects may be hormetic: this is supported by increased gene expression of *hsp-16.1* and by trend, also of *hsp-12.6*.

## Introduction

Aging is a complicated biological process that is associated with loss of physiological (e.g. stress resistance, metabolic rate, immune status), cognitive, reproductive and physical (e.g. locomotion and muscle integrity) functions which finally leads to death (Fuellen et al., 2019; Kocsisova et al., 2019). Over the past three decades, great outcomes have been achieved by aging researchers in prolonging lifespan, at least in model organisms including yeast (*Saccharomyces cerevisiae*), worms (*Caenorhabditis elegans*), and flies (*Drosophila melanogaster*), using genetic manipulations or nutritional approaches (Kirkland and Peterson, 2009). In humans, the extension of healthspan lags behind the increase of life expectancy, thus, extension of lifespan is often accompanied by the extension of time spent in ill health and reduced overall fitness (Kunugi and Mohammed Ali, 2019). Therefore, healthspan, defined as the period of life in which individuals are active and free from chronic disease, is the new target of anti-aging efforts instead of lifespan (Tissenbaum, 2015; Luyten et al., 2016). Indeed, over the past 10 years, the articles indexed in PubMed with the term “healthspan” or “health span” in the title or abstract increased dramatically (Kaeberlein, 2018).

The World Health Organization (WHO) estimated that more than 80% of the world’s population depends on herbs for their basic health care requirements (Zhang et al., 2013). Among those, Traditional Chinese Medicine (TCM) is one of the most important phytomedicine systems, which has been used in Asian countries for thousands of years (Li et al., 2019b). On May 25^th^, 2019, the WHO issued the 11^th^ revision of the International Statistical Classification of Diseases (ICD-11) (WHO, 2019) which now includes TCM as a recognized phytomedicinal approach. This reflects the contribution of TCM to the world’s healthcare, and also acknowledges the current needs for TCM (Lam et al., 2019). Now, scientific efforts are focusing on separating the harmful and placebo effects of TCM treatments from the truly beneficial effects (Break-with-Traditon, 2019). However, as discussed by Fung and Linn (2015), high quality clinical trials to determine TCM’s efficacy are still rare. More than 400 different herbs with almost 4,000 different formulas are listed in “The Encyclopedia of Traditional Chinese Medicine” (Xu et al., 2018a). Since clinical trials are expensive, protracted, and require substantial organizational efforts, the most promising TCM preparations need to be wisely chosen from that long list. The current study aimed to help with this selection process.

*Cuscuta chinensis* (*C. chinensis*) Lam. (Family: Convolvulaceae) is one of the most commonly used herbs in TCM. It contains several pharmaceutically active compounds such as phenolic compounds, polysaccharides, lignans, flavonoids and resin glycosides typical for the family Convolvulaceae (Donnapee et al., 2014; Sun et al., 2014; Wei et al., 2019a). *C. chinensis* has been reported to display anticancer, anti-apoptosis, antioxidant, anti-inflammatory, immunostimulatory, anti-osteoporotic, and hepatoprotective activities, and is also widely used in the clinical treatment of kidney yang-deficiency (Yang et al., 2011; Kang et al., 2014; Sun et al., 2014; Yang et al., 2017). *Eucommia ulmoides* (*E. ulmoides*) Oliv. (Family: Eucommiaceae) is a native tree in China (Wuyun et al., 2018), which has been used for centuries in clinical treatment according to ancient Chinese medicine books such as the Shen-nong’s Classic of Materia Medica and Compendium (Ding and Wang, 2015; Ye et al., 2019). The bark, roots, and leaves of *E. ulmoides* have been reported as a good source of highly potent medicinal compounds (Xing et al., 2019), e.g. iridoids and lignanes. Furthermore, the aqueous extracts of *E. ulmoides* bark or leaves are usually used as popular drinks in Japan, Korea, and China for hypertension treatment (Kwan et al., 2003). The antioxidant effects of *E. ulmoides* are attributed to numerous anti-oxidative flavonoids such as rutin, as well as chlorogenic-, ferulic, and caffeic acid (Xu et al., 2018b; Liu et al., 2018).

The nematode *Caenorhabditis elegans* (*C. elegans*) is a prominent model organism used in many research areas, including aging (Gruber et al., 2014; Son et al., 2019), due to numerous advantages, such as short generation time, easy and cheap maintenance, simple anatomy and completely sequenced genome (Hastings et al., 2019). Therefore, this model was used to test the hypothesis that the health-promoting effects of TCMs are based on their general anti-aging properties and are, thus, reflected in different healthspan parameters. For that purpose, heat and pathogenic stress resistance, different swim characteristics, pharyngeal pumping, body growth, fecundity, and intestinal autofluorescence were analyzed after treatment of *C. elegans* with TCM plant extracts.

This study focuses on the general health effects of *C. chinensis* and *E. ulmoides*, which were selected among seven tested TCM extracts in a preliminary screen using *C. elegans*. In addition to *C. chinensis and E. ulmoides*, the screening included plant preparations from the TCM plants *Achyranthes bidentata Blume, Astragalus membranaceus var. mongholicus (Bunge), Ligustrum lucidum* W. T.Aiton, *Wolfiporia extensa* (Peck) Ginns (syn. *Poria cocos* (Schw.) Wolf and *Schisandra chinensis* (Turcz.) Baill, all of which have been claimed to possess anti-aging properties.

## Materials and Methods

### Preparation of Extracts From TCM Used in This Study

Plants were selected by a certified TCM-specialist: Prof. Qingfei Liu (Tsinghua University, Beijing, China). They were acquired from Tong Ren Tang, Beijing (https://www.tongrentangcm.com/en/), and their identity was verified by Prof. Liu, who is trained to recognize Chinese herbs. Table 1 shows the tested organic extracts prepared by AnalytiCon Discovery GmbH (Potsdam, Germany). The extracts are stored at AnalytiCon Discovery GmbH under the batch number specified in Table 1. The dried TCM plants (20 g each) were extracted with a mixture of ^t^butyl-methyl ether (MTBE) and methanol (50:50, volume 75 ml) as well as with 100% methanol (volume 75 ml) at ambient temperature. Extraction was assisted by 15 min sonication and 2 h maceration. Both extracts of either plant were combined and dried at 45 °C by a continuous stream of air for 4 h. Thereafter, the samples were weighed and analyzed by HPLC-MS (for HPLC-MS methods and results, see Supplementary Tables S1–S3 as well as Supplementary Figures S1–S7 in the Supplementary Materials). The LC-MS chromatograms qualitatively describe the extracts, but it is not yet possible to annotate structures to the peaks.

**TABLE 1:** Traditional Chinese Medicine (TCM) extracts used in this study.

Scientific name	Common name	Family	Part of plant used	Batch number	Amount of plant used (g)	Extract amount/yield
*Achyranthes bidentata* blume	Niu Xi, ox-knee	Amaranthaceae	Root	V-22581-W-00	20	5.58 g/27.9%
*Astragalus membranaceus* (Fisch.) bunge	Huang Qi, milkvetch	Leguminosae	Root	V-22577-W-00	20	1.66 g/8.3%
*Cuscuta chinensis* Lam	Tu Si Zi, Chinese dodder	Convolvulaceae	Seeds	V-22579-W-00	20	0.93 g/4.7%
*Eucommia ulmoides* Oliv	Du Zhong, Chinese rubber tree	Eucommiaceae	Bark	V-22582-W-00	20	1.93 g/9.7%
*Ligustrum lucidum* W. T. Aiton	Nu Zhen Zi, glossy privet	Oleaceae	Fruit	V-22580-W-00	20	2.24 g/11.2%
*Poria cocos* (Schw.) Wolf	Fu Ling, Hoelen	Polyporaceae	Fruiting body	V-22587-W-00	20	0.24 g/1.2%
*Schisandra chinensis* (Turcz.) Baill	Wu Wei Zi, magnolia vine	Schisandraceae	Fruit	V-22584-W-00	20	9.12 g/45.6%

### *C. elegans* Maintenance

The wild-type *C. elegans* strain N2 (Bristol) as well as the *Escherichia coli* feeding strain OP50 were obtained from the Caenorhabditis Genetics Center (CGC) (Minneapolis, MN, United States). Nematodes were maintained according to Brenner (1974) at 22 °C on 96 mm nematode growth medium (NGM) agar plates seeded with live OP50 bacteria, which were grown at 37 °C and concentrated to OD_595_ = 5 beforehand. Synchronized and contamination-free populations were regularly generated by dissolving young adults in a 3% sodium hypochlorite solution until eggs were isolated, based on a protocol from Stiernagle (2006). The obtained eggs hatched in M9 buffer overnight, and were transferred to new NGM plates the following day.

### TCMs Treatment of *C. elegans*

The plant extracts were dissolved in DMSO at a stock concentration of 60 mg/ml and then added to the NGM agar plates as well as the OP50 bacteria at a final concentration of 30 μg/ml, while DMSO (0.05%) was used as a control in all experiments. Synchronized, untreated L4 larvae were transferred to the prepared NGM (which included 2 mg/ml carbenicillin) agar plates, followed by the addition of 100 *µ*M 5-fluorodeoxyuridine (FUdR), which prevents the development of progeny (Hosono, 1978). The nematodes were incubated on those plates at 22 °C until the respective adulthood stages, at which the following experiments were performed.

To test whether the TCMs show any antibacterial impact against *E. coli* OP50 or *P. luminescens,* the bacterial growth was determined by frequently measuring the optical density (at 595 nm) during exposure to 30 *μ*g/ml of each TCM. The results were expressed as colony-forming units (CFU/ml) according to the method described by Peñuelas-Urquides et al. (2013).

### Stress Resistance and Lifespan Assays

On the 3^rd^, 7^th^, and 12^th^ day of adulthood, worms were exposed to heat stress at 37 °C for 3 h. Thereafter, the incubation at 22 °C was continued and the number of surviving nematodes was monitored daily to determine their heat stress resistance.

For the pathogenic stress resistance assay, *Photorhabdus luminescens* (subspec. *Laumondii*, strain TT01; obtained from the Leibniz Institute DSMZ-German Collection of Microorganisms and Cell Cultures GmbH, Braunschweig, Germany) was used as a known pathogenic bacterium for *C. elegans* (Sato et al., 2014). A frozen glycerol stock of *P. luminescens* bacteria was used to inoculate 50 ml LB Lennox medium. After growing in a shaker at 28 °C for 48 h and concentrating the bacteria to a final OD_595_ = 9, the agar plates containing *P. luminescens* were prepared according to Hoinville and Wollenberg (2018): NGM plates (35 mm diameter) were seeded with *P. luminescens* mixed with *E. coli* OP50 (1:1), and incubated for an additional 48 h at 28 °C. On the 3^rd^ and 7^th^ day of adulthood, worms were shifted to these modified NGM plates containing *P. luminescens*, incubated at 22 °C, and survivors were determined daily by observing their movement after a soft touch with a platinum wire under the microscope. Each stress assay was performed blinded and repeated independently at least two times.

To determine the effect of *C. chinensis* and *E. ulmoides* on the lifespan of *C. elegans*, L4-stage synchronized worms were transferred to small NGM agar plates (see *TCMs Treatment of C. elegans*). Surviving and dead worms were counted daily until all worms had died. In addition, 100 µM quercetin hydrate (Sigma Aldrich, St. Louis, MO, United States) was used as a positive control. Approximately 75–90 worms divided over three plates were used for each treatment, and the experiment was performed three times.

### Pharyngeal Pumping Assay

The influence of *C. chinensis* and *E. ulmoides* on the pharyngeal pumping rate was measured on the 7^th^ and 12^th^ day of adulthood. About 50 worms of each exposure group and age were randomly selected and the pharyngeal pumping was determined during crawling on the OP50 seeded NGM agar by recording videos for 60 s using a digital microscope (VHX-600, Osaka, Japan) with ×500 magnification. Finally, the pumping was counted by visual inspection of the videos. The test was performed two times.

### Measuring Food Intake by Using Fluorescent Bacteria

To study the effect of *C. chinensis* and *E. ulmoides* on the food intake of *C. elegans*, the *E. coli* strain OP50-GFP (CGC, Minneapolis, MN, United States) was used according to Raizen et al., (2012). Extract-treated and control worms were transferred on the 7^th^ and 12^th^ day of adulthood to small agar plates seeded with a confluent lawn of OP50-GFP, and were allowed to feed for 15 min. Thereafter, about 25–30 nematodes per age and treatment were rinsed, washed in M9 buffer, moved to a 2% agarose pad on a glass slide, and anesthetized by 1 M NaN_3_. The worms were photographed with an Axiolab fluorescence microscope (Carl Zeiss, Jena, Germany) equipped with a GFP filter and a ProgRes C12 digital camera (Jenoptik, Jena, Germany). Mean fluorescence intensities per single worm were quantified using the CellProfiler software (Mcquin et al., 2018). The intensity values were normalized by subtracting the green autofluorescence values measured in extract-treated and control worms of the same age, fed with standard OP50.

### Intestinal Autofluorescence

The intestinal autofluorescence in extract-treated *C. elegans* was measured on the 7^th^ and 12^th^ days of adulthood according to Pincus et al., (2016). Approximately 25 individuals per age and treatment were mounted on a 2% agarose pad on a microscope glass slide, and immobilized using 1 M sodium azide. Red autofluorescence was determined and imaged using the Axiolab fluorescence microscope (Carl Zeiss, Jena, Germany) with a TRITC filter set (excitation: 546 nm; emission: 600 nm), and equipped with a ProgRes C12 digital camera (Jenoptik, Jena, Germany). Mean fluorescence intensities per nematode were quantified densitometrically using the CellProfiler software (Mcquin et al., 2018).

### Swimming Behavior

Analysis of the swimming behavior of wild type *C. elegans* was carried out using the CeleST software (Restif et al., 2014). On the 7^th^ and 12^th^ day of adulthood, five worms each were transferred to wells with a depth of 0.5 mm and a diameter of 10 mm on a microscope slide, which were filled with M9 buffer and covered by a cover slip to facilitate visualization. Then, 60-s videos were recorded per well and ≥50 nematodes were used per treatment and age. After isolating every second frame of the videos, and applying the greyscale and invert mode via Adobe Photoshop, the wave initiation rate, activity index, brush stroke and body wave number were determined with the CeleST software.

### Body Size and Fecundity Measurements

The size of treated and untreated worms was measured by transferring them on the 7^th^ and 12^th^ day of adulthood to a 2% agarose pad on a glass slide, and anesthetizing them by using 1 M sodium azide. The bright field image of each animal was captured using a microscope (Carl Zeiss, Jena, Germany) equipped with a digital camera (ProgRes C12, Jenoptik, Jena, Germany). The body size of at least 40 nematodes per treatment was measured by determining the number of covered pixels per body with the CellProfiler software.

The effect of *C. chinensis* and *E. ulmoides* on fecundity was determined by counting the daily and total offspring per nematode. Ten synchronized L4 hermaphrodites, which were treated with the tested plant extracts since L1 larval stage, were placed individually to treatment and control plates at 22 °C without the reproductive inhibitor FUdR. Every 24 h, each single worm was transferred to a fresh plate until day 3 of adulthood. The hatched eggs were allowed to develop to L2 or L3 larvae and then the total offspring produced by each individual was counted per day and finally summed up.

### Quantitative Real-Time PCR

About 3,000 worms per sample were collected on ice on the 12^th^ day of adulthood, washed with M9-buffer, transferred to lysis tubes containing ceramic beads and 100 µl Lysis Solution RL (innuSPEED Tissue RNA Kit, Analytik Jena, Germany) and then ground thoroughly in a homogenizer (speedMill plus, Analytik Jena, Germany). Total RNA was extracted according to the manufacturer’s protocol (innuSPEED Tissue RNA Kit, Analytik Jena, Germany) with subsequent DNAse treatment (TURBO DNA-free Kit, Invitrogen, Carlsbad, CA, United States). Gel-electrophoresis and NanoDrop measurements were used to determine the quality and quantity of the RNA samples. cDNA synthesis was performed with the Script cDNA Synthesis Kit (Jena Biosciences, Jena, Germany) using 1 µg of total RNA per sample. The Quantitative Real-Time PCR (qPCR) analysis was conducted with the Luna Universal qPCR Kit (New England BioLabs, Frankfurt a.M., Germany) and the MyiQ Single Color RT-PCR Detection System (Bio-Rad, Hercules, CA, United States). Each sample was measured in three biological replicates, and each biological replicate was measured in technical triplicates. The fold changes were calculated according to Pfaffl (2001) and *act-1* and *cdc-42* were used as reference genes for normalization. The primer sequences, annealing temperatures and efficiencies as well as the qPCR cycle protocol are given in Supplementary Tables S4, S5.

### Statistical Analysis

Statistical analysis was carried out using the one-way ANOVA test followed by the Bonferroni’s multiple comparison test (https://astatsa.com/OneWay_Anova_with_TukeyHSD/) or a log-rank test via the Online Application for Survival analysis OASIS 2 (Han et al., 2016) with subsequent Bonferroni correction. Data are displayed as mean ± SEM (standard error of the mean). Changes were considered statistically significant if their *p*-value was *(*p* < 0.05), **(*p* < 0.01), ***(*p* < 0.001) or ****(*p* < 0.0001).

## Results

### *E. ulmoides* and *C. chinensis* Increased Heat Stress Resistance of *C. elegans*

**FIGURE 1:**
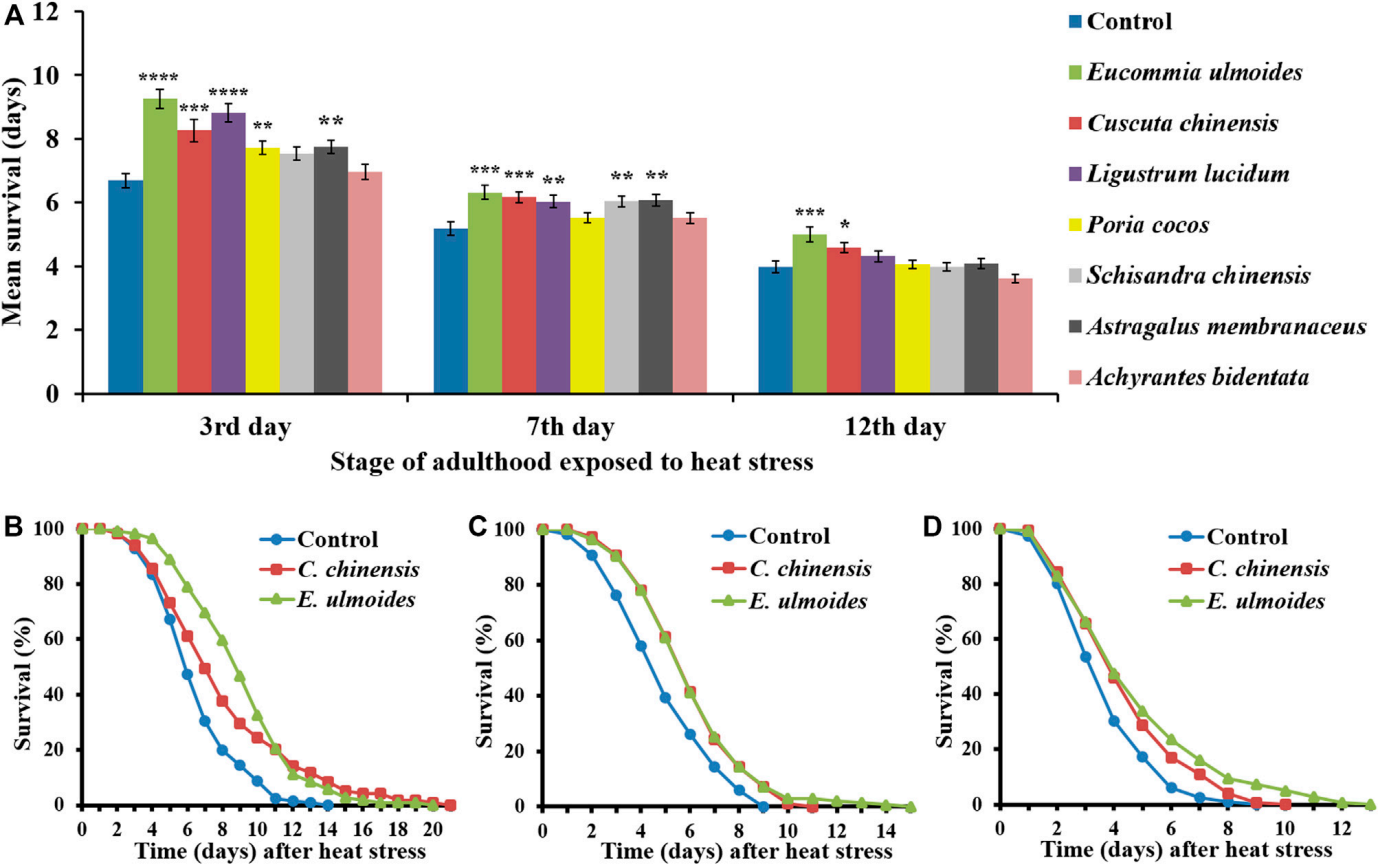
Heat stress resistance of *C. elegans* in the presence of different TCM extracts. *C. elegans* was treated with seven different TCM plant preparations (30 μg/ml), and DMSO as control, starting at the L4 stage. Heat stress (37 °C for 3 h) was applied at different ages of adulthood (3^rd^, 7^th^ and 12^th^ day), and the survival after stress was monitored in the different treatment groups **(A)**. The values represent the mean survival ± SEM from three biological replicates. Significant differences were determined by a log-rank test and Bonferroni correction, with *p < 0.05; **p < 0.01; ***p < 0.001 and ****p < 0.0001. In addition, representative survival curves are shown for nematodes treated with *E. ulmoides* and *C. chinensis* extracts, which were heat-stressed on the 3^rd^ **(B)**, 7^th^ **(C)** and 12^th^ **(D)** day of adulthood.

To determine the effect of TCM extracts on heat stress resistance of *C. elegans,* extract-treated and control nematodes were exposed to a heat shock (37 °C for 3 h) at different ages of adulthood (3^rd^, 7^th^, and 12^th^ day). Representative survival curves as well as mean survival following the heat stress condition are presented in Figure 1. The application of several extracts significantly increased the stress resistance of younger worms to heat shock. However, among the seven extracts, only *E. ulmoides* and *C. chinensis* were able to improve the survival in older (12-days) nematodes (Figures 1A,D). In contrast, the organic extract from *Achyrantes bidentata* was not able to provoke any changes in survival rates, neither in young nor in old nematodes.

The mean survival of control animals after heat shock on the 3^rd^, 7^th^, and 12^th^ day of adulthood amounted to 6.7, 5.2, and 4.0 days, respectively (Table 2), reflecting the general aging-dependence of the heat stress resistance parameter. The incubation with *C. chinensis* caused a survival increase at the aforementioned ages to 8.3, 6.2, and 4.6 days, respectively. Interestingly, application of *E. ulmoides* exhibited the strongest overall effect by boosting the nematodes’ survival to 9.3, 6.3 and 5.0 days, respectively. Figures 1B–D revealed that not only the mean lifespan was increased by these two extracts, but also the maximum lifespan (Table 2).

**TABLE 2:** Survival after heat stress in the presence and absence of TCM extracts.

Start of stress	Treatment	Mean lifespan		Min. [days]	Med. [days]	Max. [days]	*n*	*p-*value
		Days ± SE	%					
3rd day of adulthood	Control	6.69 ± 0.22	100.0	4.54	5.88	14	125	
	*E. ulmoides*	9.26 ± 0.30	138.4	6.42	8.80	22	107	<0.0001
	*C. chinensis*	8.26 ± 0.36	123.5	4.85	6.96	21	119	0.0004
7th day of adulthood	Control	5.19 ± 0.21	100.0	3.09	4.45	9	120	
	*E. ulmoides*	6.32 ± 0.22	121.8	4.20	5.57	15	139	0.0006
	*C. chinensis*	6.17 ± 0.17	118.9	4.20	5.57	11	153	0.0003
12th day of adulthood	Control	3.99 ± 0.18	100.0	2.20	3.17	9	117	
	*E. ulmoides*	5.00 ± 0.24	125.3	2.48	3.88	13	140	0.0006
	*C. chinensis*	4.58 ± 0.16	114.8	2.51	3.80	10	147	0.0114

n, total number of worms; SE, Standard error; Min/Med/Max, minimum/median/maximum lifespan (days until deaths in population reached 25%/50%/100%). The p-values express significance using a log-rank test with subsequent Bonferroni correction.

### *E. ulmoides* and *C. chinensis* Improved the Resistance to *Photorhabdus luminescens*

*Photorhabdus luminescens* is able to infect *C. elegans* and consequently increases the death rate (Sato et al., 2014; Hoinville and Wollenberg, 2018). In this study, the ability was investigated of TCM extracts to counteract the pathogen-induced mortality in *C. elegans* exposed to the *P. luminescens* strain TT01 starting on the 3^rd^ and 7^th^ day of adulthood. Since the ingestion of pathogens (essential for infection) cannot be guaranteed in old nematodes due to the drastically decreased pharyngeal pumping frequency, no pathogen resistance assay was started on the 12^th^ day of adulthood.

**FIGURE 2:**
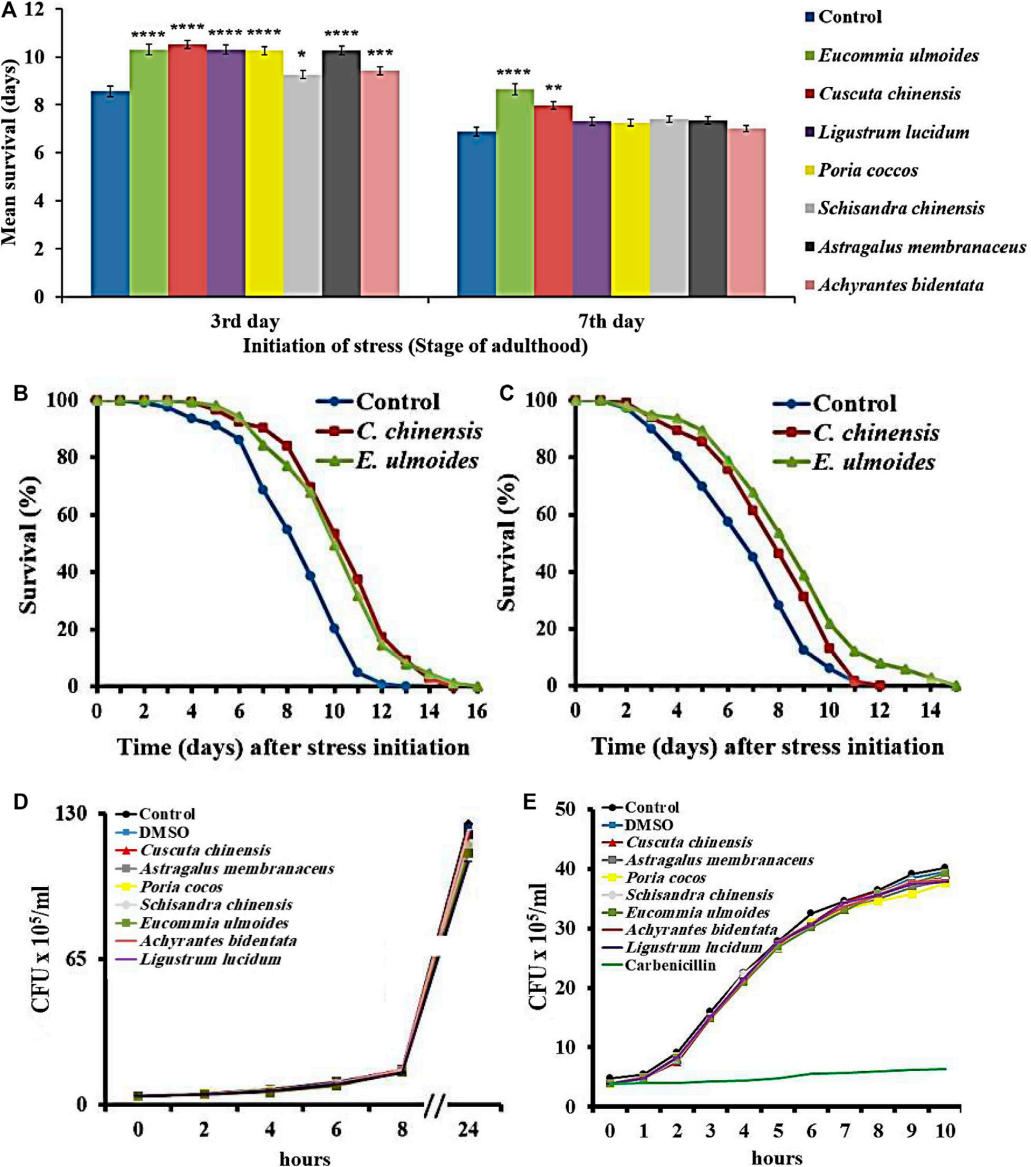
Survival of *C* *. elegans* during exposure to the pathogenic bacterium *P. luminescens.*C*. elegans* was treated with seven different TCM plant preparations (30 *μ*g/ml), and DMSO as control, starting at the L4 stage **(A)** Pathogenic stress due to *P. luminescens* exposure was initiated on the 3^rd^ or 7^th^ day of adulthood, and the survival during stress was monitored in the different treatment groups. The values represent the mean survival ± SEM from three biological replicates. Significant differences were determined by log-rank test and Bonferroni correction with *p < 0.05; **p < 0.01; ***p < 0.001 or ****p < 0.0001. In addition, survival curves are shown for nematodes treated with *E* *. ulmoides* and *C* *. chinensis* extracts, which were exposed to the pathogen starting on the 3^rd^ **(B)** and 7^th^ **(C)** day of adulthood **(D)** The growth of *P. luminescens* and **(E)** *E* *. coli* OP50 was monitored during extract exposure and the colony-forming units per time point are shown.

As expected, the pathogen sensitivity increases with age, underlining the suitability of pathogen stress assays in aging research (Figure 2A). All extracts were able to prolong the survival during pathogen exposure when starting on the 3rd day of adulthood (Figure 2A). However, only *E. ulmoides* and *C. chinensis* caused in addition a survival advantage in middle-aged nematodes (Figures 2A–C). In detail, *E. ulmoides* and *C. chinensis* could prolong the mean survival of *C. elegans* infected on the 3^rd^ day of adulthood to 10.3 and 10.5 days, respectively, compared to 8.6 days for untreated worms. For nematodes infected on the 7^th^ day of adulthood, *E. ulmoides* and *C. chinensis* extended the mean survival by 26 and 16%, respectively, but only *E. ulmoides* was able to prolong the maximum lifespan in both assays (Table 3). To exclude simple pathogen-inhibiting effects by the extracts as the underlying reason for increased resistance, the growth of liquid *P. luminescens* cultures was observed during extract treatment (30 *μ*g/ml). All treated cultures were able to grow in the same manner as the control (no added substance) or the DMSO solvent control (Figure 2D). Thus, antibacterial effects did not play a role in the observed pathogenic resistance. Moreover, the tested extracts also had no effect on the growth of OP50 (Figure 2E).

**TABLE 3:** Nematode survival during *P. luminescens* exposure in the presence and absence of TCM extracts.

Start of stress	Treatment	Mean lifespan		Min. [days]	Med. [days]	Max. [days]	*n*	*p-*value
		Days ± SE	%					
3rd day of adulthood	Control	8.56 ± 0.20	100.0	6.64	8.3	13	124	
	*E. ulmoides*	10.30 ± 0.18	120.3	8.22	9.97	16	174	<0.0001
	*C. chinensis*	10.52 ± 0.19	122.9	8.62	10.22	15	144	<0.0001
7th day of adulthood	Control	6.88 ± 0.19	100.0	4.52	6.61	12	153	
	*E. ulmoides*	8.65 ± 0.23	125.7	6.35	8.24	15	142	<0.0001
	*C. chinensis*	7.97 ± 0.20	115.8	6.05	7.75	12	132	0.0013

*n*, total number of worms; SE, Standard error; Min/Med/Max, minimum/median/maximum lifespan (days until deaths in population reached 25%/50%/100%). The p-values express significance using a log-rank test with subsequent Bonferroni correction.

Based on their effectiveness in the above shown stress resistance assays, further tests focused solely on treatments with *E. ulmoides* and *C. chinensis* extracts.

### *C. chinensis* and *E. ulmoides* Enhanced Longevity

**FIGURE 3:**
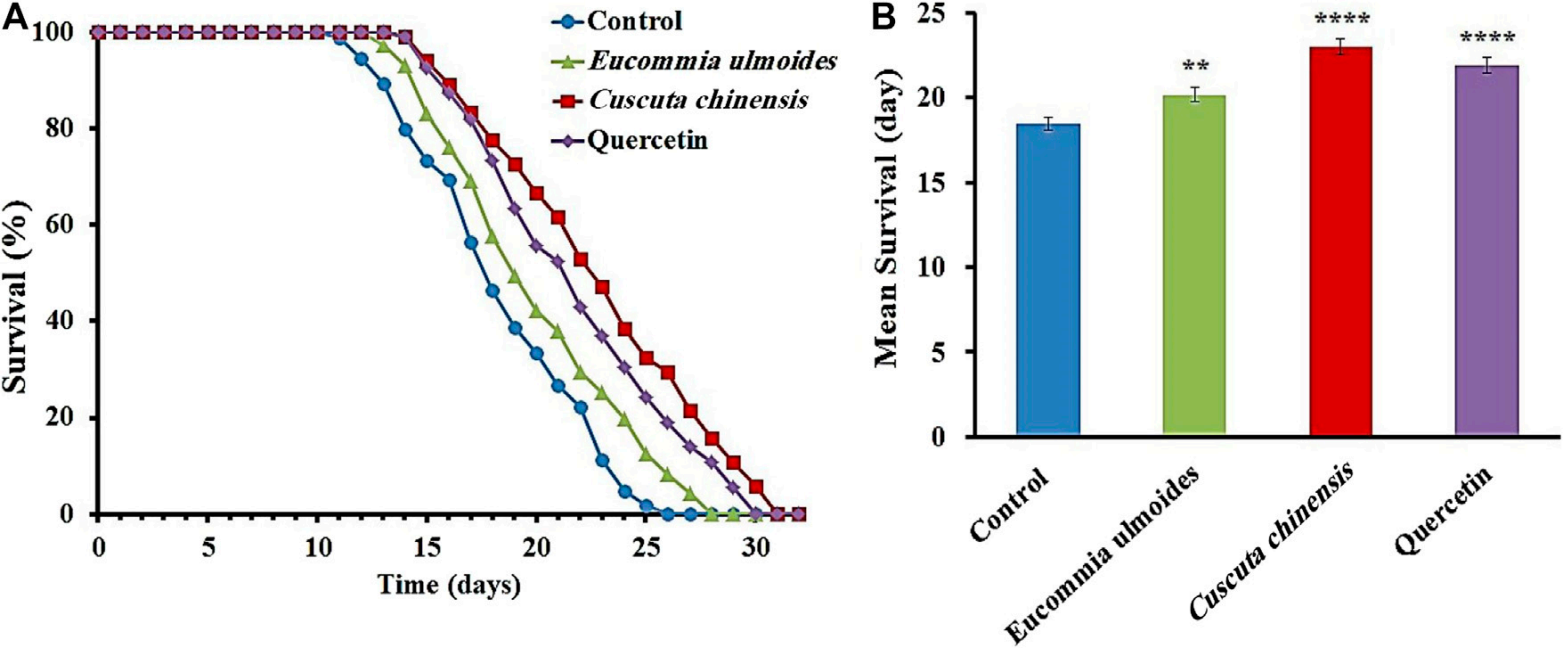
Effects of *C. chinensis* and *E. ulmoides* extracts on the lifespan of wild type *C. elegans.* **(A)** Representative survival curves as well as **(B)** the mean lifespan ± SEM from three biological replicates are shown; 100 µM quercetin was used as a positive control. Significant differences were determined by a log-rank test and subsequent Bonferroni correction with *p < 0.05; **p < 0.01; ***p < 0.001 or ****p < 0.0001.

In addition to the survival under stressful conditions, the lifespan was also monitored under standard laboratory conditions. *C. elegans* exposed to *E. ulmoides* or *C. chinensis* extract featured a significant increase of the mean lifespan, as shown in the representative survival curves (Figure 3). *C. chinensis* caused the highest increase of the mean lifespan (24%), while *E. ulmoides* increased it by only 9%. Quercetin was used as a positive control and its increase in mean lifespan by 19% ranged between the effects from *E. ulmoides* and *C. chinensis*. Furthermore, all treatments were able to increase the minimum, median and maximum lifespan (Table 4).

**TABLE 4:** Effect of *C. chinensis* and *E. ulmoides* on the lifespan of *C. elegans*.

Treatment	Mean lifespan		Min. [days]	Med. [days]	Max. [days]	*n*	*p-*value
	Days ± SE	%					
Control	18.48 ± 0.4	100	14.71	17.61	26.00	275	
*E. ulmoides*	20.19 ± 0.43	109.28	16.36	19.07	28.00	265	0.0065
*C. chinensis*	22.98 ± 0.47	124.39	18.50	22.50	31.00	225	<0.0001
Quercetin	21.90 ± 0.46	118.52	17.81	21.22	30.00	246	<0.0001

n, total number of worms; SE, Standard error; Min/Med/Max, minimum/median/maximum lifespan (days until deaths in population reached 25%/50%/100%). The p-values express significance using a log-rank test with subsequent Bonferroni correction.

### *C. chinensis* Increased the Pharyngeal Pumping Frequency and Food Intake of *C. elegans*

**FIGURE 4:**
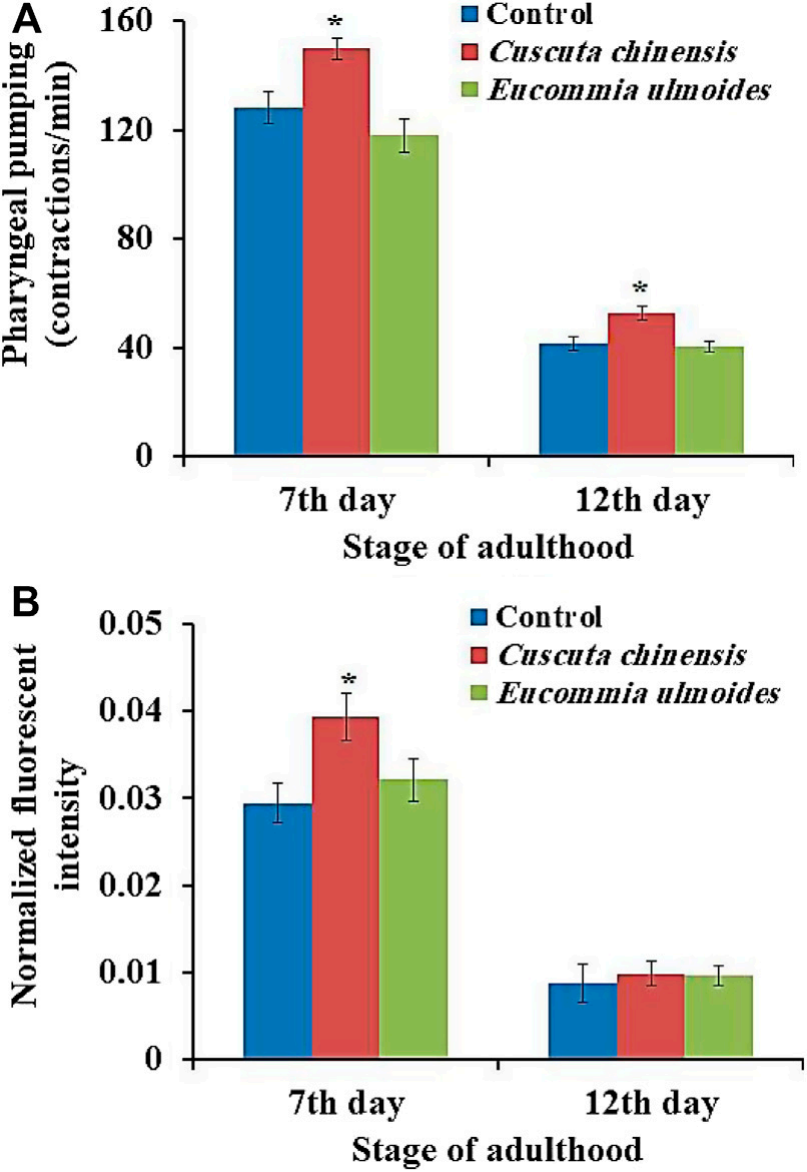
Influences of *E* *. ulmoides* and *C* *. chinensis* extracts on **(A)** pharynx pumping rate and **(B)** intake of the *E. coli* strain OP50-GFP. Synchronized L4 larvae were transferred to NGM plates in the absence (control) or presence (30 μg/ml) of the respective plant extract. On the 7^th^ and 12^th^ days of adulthood, the pharyngeal pumping rate of individuals (*n* = 50) was measured under a stereoscopic microscope for 60 s. The results represent mean ± SEM and significant changes to the control are considered *(*p* < 0.05), **(*p* < 0.01), ***(*p* < 0.001) or ****(*p* < 0.0001) according to one-way ANOVA and post-hoc Bonferroni test.

*C. elegans* feeds by a cycle of contraction and relaxation of the pharyngeal muscles, which can be monitored by up and down movement of the grinder. The pharyngeal pumping rate in *C. elegans* determines the food intake and mirrors the general fitness status of the organism. The pumping was measured in wild-type adult animals on the 7^th^ and 12^th^ days of adulthood in the presence or absence of the plant extracts. The pumping frequencies of the pharynx of untreated nematodes (control) were about 128 and 42 pumps per minute on the 7^th^ and 12^th^ days of adulthood, respectively. *C. chinensis* exposure led to a significant increase of the pharyngeal pumping rate, with 150 and 53 pumps per minute on the 7^th^ and 12^th^ day of adulthood, respectively. On the other hand, the treatment with *E. ulmoides* did not lead to any frequency changes in either age class (Figure 4A).

In addition, food intake was evaluated by measuring the intensity of fluorescence in the intestinal lumen after feeding the worms with the *E. coli* strain OP50-GFP for 15 min. The analysis of fluorescent intensity revealed that the *C. chinensis* extract increased the amount of OP50-GFP bacteria in the worms’ intestine significantly by 34% on the 7^th^ day and non-significantly by 12% on the 12^th^ day of adulthood compared with the control group. The treatment with the *E. ulmoides* extract resulted in non-significant increase in the food uptake by only 9% on the 7^th^ and 12^th^ day of adulthood, respectively (Figure 4B).

### *C. chinensis* Reduced the Accumulation of Autofluorescent Material

**FIGURE 5:**
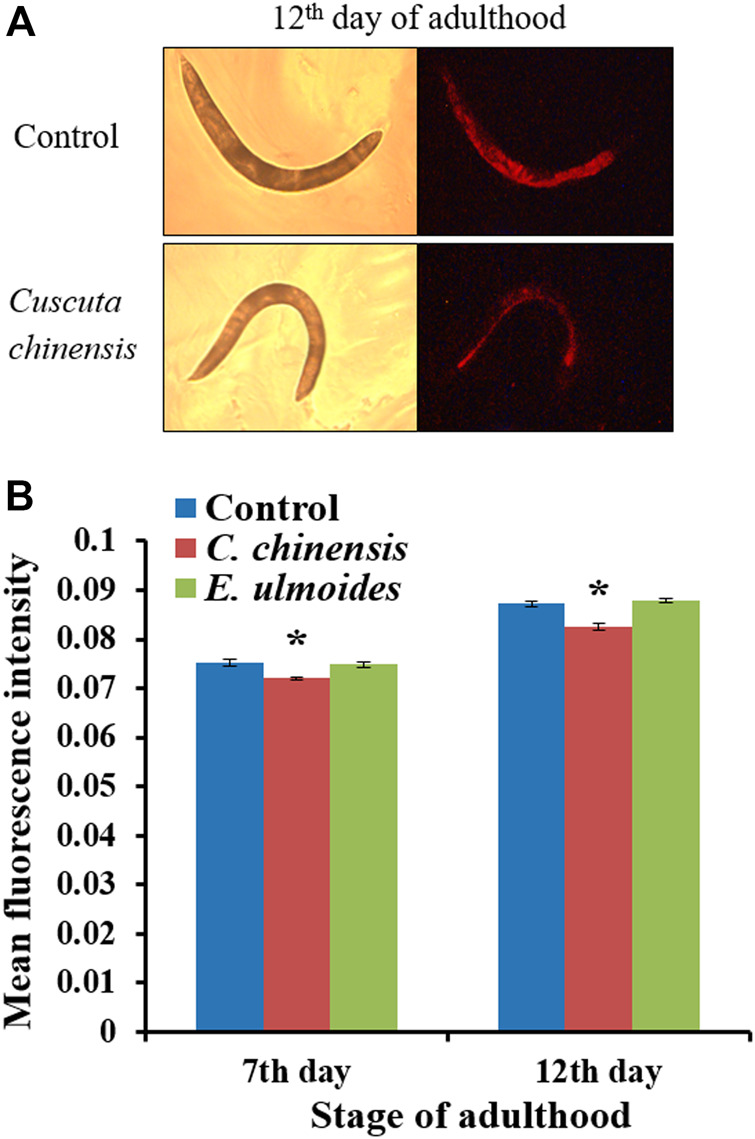
Red autofluorescence of wild-type *C. elegans* treated with 30 *μ*g/ml plant extract. On the 7^th^ and 12^th^ days of adulthood, treated and untreated nematodes were photographed in bright field as well as under red fluorescent light. **(A)** Representative control and *C. chinensis*-treated nematode on the 12^th^ day of adulthood **(B)** The fluorescence intensity of individuals was determined by densitometric analysis using the CellProfiler software. The results are the average intensity for two independent experiments (*n* ≥ 25 per experiment). The intensity was represented as mean ± SEM and the significant changes to the control are considered *(*p* < 0.05), **(*p* < 0.01), ***(*p* < 0.001), or ****(*p* < 0.0001) according to one-way ANOVA and post-hoc Bonferroni test.

Autofluorescent material accumulates over time in cells and tissues with low turnover (Terman and Brunk, 2006), and is often used as a marker of aging in *C. elegans* (Pincus and Slack, 2010). We evaluated the intestinal autofluorescent material accumulation, as a marker of aging, of *C. elegans* on the 7^th^ and 12^th^ days of adulthood. *C. elegans* treated with *C. chinensis* showed significantly reduced autofluorescence intensities to 96.1 and 94.2% on the 7^th^ and 12^th^ days of adulthood, respectively, compared with the untreated group (100%). In contrast, treatment with *E. ulmoides* had no impact on red fluorescence in either age group (Figure5).

### *C. chinensis* Enhanced Locomotor Fitness in Liquid Media

**FIGURE 6:**
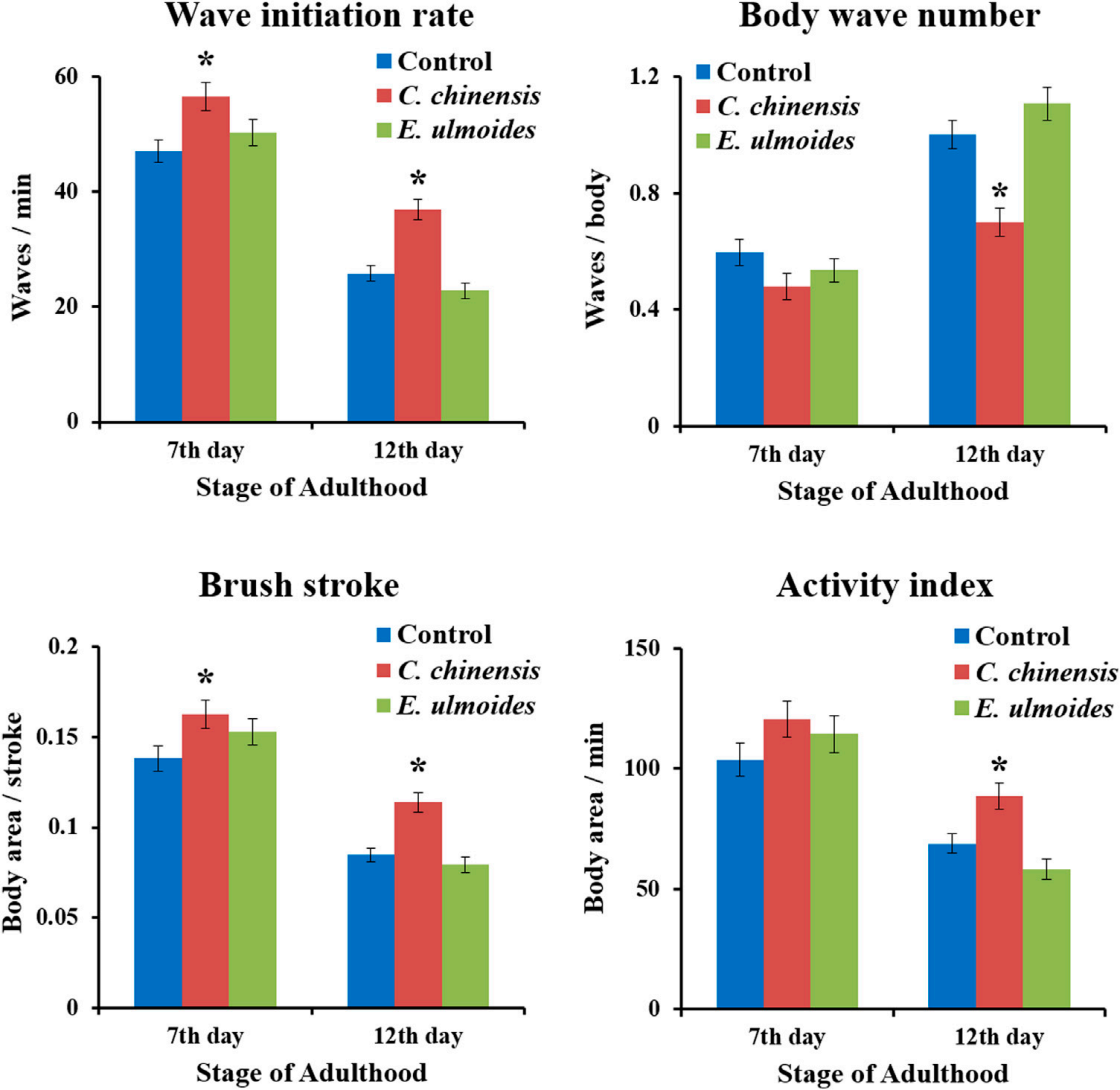
Swim performance of *C. elegans* after *C. chinensis* and *E. ulmoides* treatment. Wave initiation rate, body wave number, brush stroke and activity index were determined on the 7th and 12th day of adulthood. Error bars are the standard error of the mean (SEM) and one bar represents *n* ≥ 50 from two independent trials. Statistical significance was determined according to one-way ANOVA and post-hoc Bonferroni test as *(*p* < 0.05), **(*p* < 0.01), ***(*p* < 0.001), or ****(*p* < 0.0001).

We investigated the ability of *C. chinensis* and *E. ulmoides* to improve the nematode’s swimming behavior on the 7^th^ and 12^th^ days of adulthood by measuring four parameters: wave initiation rate, activity index, brush stroke, and body wave number. Indeed, the data obtained in the current study verify the age-dependence of all selected swim parameters when comparing the 7^th^ and 12^th^ days of adulthood (Figure 6). On the 7^th^ day of adulthood, *C. chinensis* could increase the wave initiation rate to 56.6 waves per minute comparing with the control (47 waves per minute). Furthermore, the brush stroke was also enhanced by *C. chinensis* and at least a positive, albeit not statistically significant, trend is visible for the other two parameters. Interestingly, *C. chinensis* caused stronger effects on the 12^th^ day of adulthood with statistically significant improvements in all measured parameters. By contrast, *E. ulmoides* did not produce any statistically significant difference on the swimming performance of *C. elegans* (Figure 6).

### Only Slight Impacts of *E. ulmoides* and *C. chinensis* on Body Size and Reproduction

**FIGURE 7:**
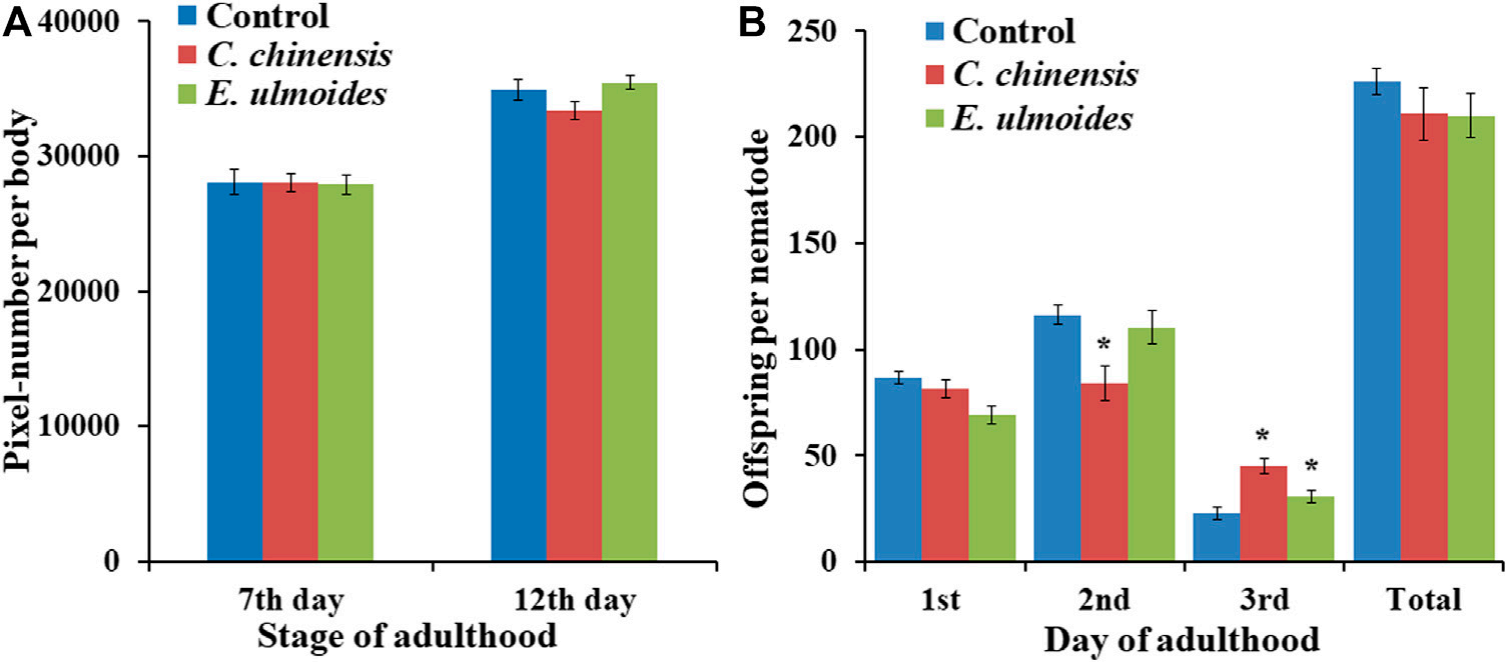
Impact of *E. ulmoides* and *C. chinensis* on body size and reproduction of *C. elegans*. **(A)** Body size of *C. elegans* on the 7^th^ and 12^th^ day of adulthood after TCM treatment (*n* ≥ 25 per experiment). **(B)** The number of offspring per day and in total during exposure to TCM treatment (*n* = 27–30 nematodes per treatment). The results display the average size for three independent experiments and error bars represent the SEM. Statistically significant differences to the control are considered with *p < 0.05 according to one-way ANOVA and post-hoc Bonferroni test.

In the current study, we measured the effect of *E. ulmoides* and *C. chinensis* on the growth as well as reproductive fitness of *C. elegans.* Indeed, after measuring the body area of treated and untreated worms on the 7^th^ and 12^th^ days of adulthood, the results showed that none of the tested TCM preparations had a significant effect on the body size of *C. elegans*, whereas a slight reducing trend is visible on the 12^th^ day of adulthood in *C. chinensis* treated worms (Figure 7A). Moreover, exposure to *E. ulmoides* and *C. chinensis* did not significantly influence reproductive fitness in terms of the total number of progenies produced per individual (Figure 7B). However, we found that *E. ulmoides* and *C. chinensis* delayed the reproduction by decreasing the number of offspring on the first (*E. ulmoides*; not significant) or second (*C. chinensis*) day, respectively (Figure 7B). Interestingly, the extract treated worms led to a significant increase of the offspring on the third day of observation. Our results revealed that the selected TCM preparations increased the healthspan of *C. elegans* without significantly impairing overall growth, but with a slight impact on the timing of reproduction, which may reflect a delay in reproductive (ovarian) aging.

### *C. chinensis* Treatment Increased the Transcription of Heat Shock Protein *Hsp-16.1*

**FIGURE 8:**
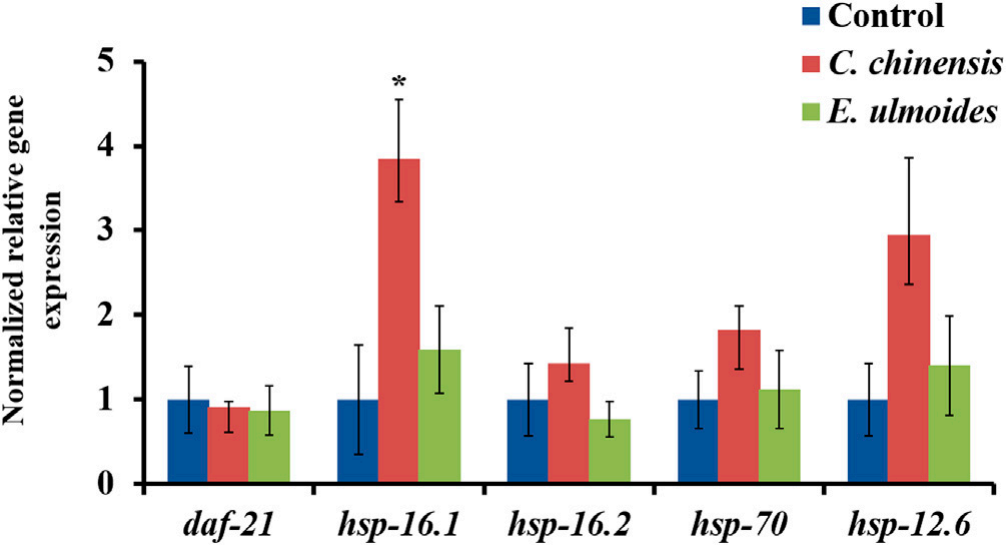
Effect of *C. chinensis* and *E. ulmoides* treatment on the gene expressions levels of heat-shock proteins at the 12^th^ day of adulthood. Gene expression was determined via qPCR, and expression values were calculated according to Pfaffl (2001). Gene expression values of the reference genes *act-1* and *cdc-42* were used to normalize the data. The graph shows the mean of three biological replicates. The error bars show the standard error of the mean (SEM) and statistical significance was determined according to one-way ANOVA and post-hoc Bonferroni test with *(*p* < 0.05).

To get a first insight into the molecular mechanism of the observed healthspan effects, the gene expression for five heat shock proteins, namely *daf-21* (*hsp-90*), *hsp-16.1*, *hsp-16.2*, *hsp-70*, and *hsp-12.6*, was analyzed in extract-treated nematodes on the 12^th^ day of adulthood. The exposure to *C. chinensis* significantly upregulated the expression of *hsp-16.1* by 3.8-fold (Figure 8). Moreover, the expression of *hsp-12.6* also increased after *C. chinensis* treatment by 2.9-fold; however, due to the high variance and limited sample number, the significance threshold was not reached. Interestingly, treatment with *E. ulmoides* revealed no significant changes in the expression level of any tested genes.

## Discussion

An organism’s lifespan is inevitably accompanied by the aging process, which involves functional decline, a steady increase of a plethora of chronic diseases, and ultimately death. Thus, it has been an ongoing dream of mankind to improve healthspan and extend life. In the last century, developed countries have profited from medical advances, improvements in public healthcare systems, and better living conditions derived from their socioeconomic power to achieve a remarkable increase in life expectancy. However, according to the WHO, age itself remains the greatest risk factor for all major life-threatening disorders, and the number of people suffering from age-related diseases is anticipated to almost double over the next two decades. The fact that healthspan has not increased at the same pace as lifespan is a source of grave concern (de Cabo et al., 2014). Attempts for living longer and healthier have attracted the attention of both the public and researchers. One interesting method for inducing a longer and healthier life is dietary supplementation with TCM. This study evaluated the anti-aging properties and health-related impacts of *C. chinensis* and *E. ulmoides* using *C. elegans*. Indeed, various nutritional and pharmacological interventions with *C. chinensis* and *E. ulmoides* have been previously shown. For a detailed overview of the bioactivities reported for *C. chinensis* and *E. ulmoides* see Tables 5, 6.

**TABLE 5:** Pharmacological effects and bioactivities of *Eucommia ulmoides* Oliver.

Bioactivity	Model system or organism	Plant parts	References
Anti-apoptosis	Glioblastoma cell lines	Leaf	Wang et al. (2019)
Anti-diabetic action	Rats	Leaf	Jin et al. (2010)
Anti-hypertensive effect	Rats	Bark	Luo et al. (2010)
Anti-inflammatory activity	Microglial cell culture and battery of assay models	Bark	Kim et al. (2009), Kwon et al. (2016)
Anti-obesity	Human hepatoma HepG2 cells and mice	Leaf	Hirata et al. (2011), Hao et al. (2016)
Antioxidant properties	Human SH-SY5Y neuroblastoma cells	Bark	Kwon et al. (2012)
Anti-steatosis activity	Rats	Leaf	Lee et al. (2019)
Management of hyperlipidemia	Rats	Leaf	Horii et al. (2010), Kobayashi et al. (2010)
Neuroprotective effects	Rats	Bark and leaf	Zhou et al. (2009), Kwon et al. (2011)
Regulation of hepatic lipotoxicity	Rats	Bark	Lee et al. (2014)
Reversed hypertensive vascular remodeling	Rats	Bark	Gu et al. (2011)
Treatment of cognitive deficits	Mice	Bark	Kwon et al. (2013)
Treatment of osteoporosis	Rats	Bark	Zhang et al. (2009), Zhang et al. (2014), Qi et al. (2019)

**TABLE 6:** Pharmacological effects and bioactivities of *Cuscuta chinensis* Lam.

Bioactivity	Model system or organism	Plant parts	References
Anti-aging effect	Rats	Seeds	Li et al. (2013)
Anti-apoptosis	Rats and mice	Seeds	Sun et al. (2014), Qin et al. (2019), Wei et al. (2019b)
Anticancer activity	Cell culture	Erial parts	Ghazanfari et al. (2013), Jafarian et al. (2014)
Anti-hepatofibrosis	Rat	Seeds	Kim et al. (2017)
Anti-inflammatory activity	Rats and cell culture	Seeds	Kang et al. (2014), Ding et al. (2019)
Anti-nociceptive and anti-inflammatory	Mice	Seeds	Liao et al. (2014)
Anti-osteoporotic	Osteoblastic cells	Seeds	Yang et al. (2011)
Effect of reversing kidney-yang deficiency	Rats	Seeds	Yang et al. (2017)
Improves sperm quality and reduces oxidative damage in testis and epididymis	Mice	Seeds	Cui et al. (2019)
Inhibition of melanogenesis	Zebrafish larvae	Seeds	Wang et al. (2014)
Modulation of oxidative stress-induced apoptosis	Cell culture	Seeds	Gao et al. (2013)
Regulation of urine concentration and renal functions	Rats	Seeds	Shin et al. (2011)
Relaxation effect	Rabbit	Seeds	Sun et al. (2013)
Treatment of kidney failure	Rats	Seeds	Zhang et al. (2018a)

### Hormesis: A Possible Explanation for Age-dependent Effects of TCM-Mediated Heat Stress Resistance

In this study, the tested TCMs showed positive impact on heat stress resistance of *C. elegans* exposed to a heat shock (37 °C for 3 h) at different ages of adulthood (3^rd^, 7^th^, and 12^th^ days). The beneficial effect of TCMs on heat stress resistance is consistent with other studies in *C. elegans* that showed enhancement of both, thermal tolerance and lifespan by other TCM-derived extracts, such as extracts from *Zanthoxylum armatum* DC. and Salvia miltiorrhiza Bunge (Pandey et al., 2019; Zhang et al., 2019). In addition, previous studies reported that quercetin, a common flavonoid found in TCMs, increased the tolerance to thermal stress, which was applied to the worms by heating them to 37 °C (Liu et al., 2016) or 35 °C (Saul et al., 2008). Thus, the enhancement of heat stress resistance by TCM could be based on a bioactive compound common to many of these medicinal plants that may regulate stress-signaling pathways. Alternatively, these compounds may act themselves as chemical chaperones that stabilize protein conformation and promote a general cellular stress response as described by Benedetti et al. (2008).

We could show that the ability of the TCMs to enhance stress resistance depends on the age of nematodes during test-performance. But what do the different tested adult-stages stand for? The 3^rd^ day of adulthood reflects the time when the nematode is reproductive, very active and healthy; thus, symptoms of aging are not readily apparent. However, Kauffman et al. (2010) could show that learning and memory abilities are surprisingly impaired already at this young stage. On the 7^th^ day of adulthood, reproduction came to a halt, movements are slower, stress resistance is decreased and long-term memory vanishes (Kauffman et al., 2010); thus, the effects of aging become visible. On the 12^th^ day of adulthood, aging strongly emerges with the appearance of immobile nematodes, high stress-sensitivity, morphological abnormalities and deaths. Since the general anti-aging properties of TCMs are the main focus of this paper, results from the 7^th^ and especially 12^th^ day of adulthood were deemed more relevant.

The question arises why so many extracts are active in young, but not in old nematodes? The answer could be related to the hormesis effect, which is the biphasic response to low and high doses of numerous chemical, biological, or physical insults (Kendig et al., 2010). Several studies suggested the hormesis effect as the main mechanism underlying the beneficial effects of natural extracts and polyphenols (Saul et al., 2011; Martel et al., 2019), which is sometimes also called more specifically “xenohormesis” (Suter and Lucock, 2017). It is known that hormesis is dependent on the timing and duration of treatment (Tyne et al., 2015) and that the adaptive response to (mild) stressors is less active in aged individuals (Pomatto and Davies, 2017). Therefore, the lack of activity for most of the tested extracts in old nematodes could be due to their hormetic action being less efficient in old nematodes and/or in long-term treatment scenarios. For the few plant preparations that show positive effects in older nematodes, a mechanism distinct from hormesis may be at work.

To test whether hormesis might also explain the beneficial effects of *C. chinensis* and *E. ulmoides* in old nematodes, the gene expression of five *hsp*-genes was determined in treated and untreated animals on the 12^th^ day of adulthood. Molecular chaperones were shown to be implicated in adaptive stress response and hormesis (Lakshmi et al., 2020), and especially heat shock proteins and their chaperone-activities play a fundamental role in hormesis in *C. elegans* (Zhao and Wang, 2012). Therefore, *daf-21* (*hsp-90*) was specifically selected due to its implication in pathogenic stress resistance (JebaMercy et al., 2016; Balasubramaniam et al., 2019), in the stabilization of the ageing-relevant nutrient-sensor SIR-2.1 (Nguyen et al., 2018) and the DAF-16-mediated lifespan regulation (Somogyvári et al., 2018). Furthermore, in response to hormetic life- and healthspan prolonging treatments, the up-regulation of *hsp-16.1* (Yanase et al., 1999; Lin et al., 2019), *hsp-16.2* (Olsen et al., 2006; Hartwig et al., 2009), as well as *hsp-70* and *hsp-12.6* (Pietsch et al., 2011) was described. In our study, only *hsp-16.1* was differentially expressed and a clear, but not statistically significant, trend was observed for *hsp-12.6*. Both genes were up-regulated after chronic *C. chinensis* exposure, whereas no response was detected after *E. ulmoides* treatment. This underlines the different mode of action of these two extracts, and suggests a possible hormetic action of the *C. chinensis* extract.

### Increased Pathogen Resistance due to a TCM-Mediated Boost of the Immune Response?

*P. luminescens* has been reported as a nematode-symbiotic bacterium that causes a lethal intestinal infection in *C. elegans* and consequently reduces the worm’s developmental, survival and reproductive capacity (Sato et al., 2014; Hoinville and Wollenberg, 2018). In this study, the ability was investigated of TCM extracts to counteract the pathogen-induced mortality in *C. elegans* exposed to *P. luminescens*. *E. ulmoides* and *C. chinensis* increased the pathogenic resistance of *C. elegans* against *P. luminescens* by extending the survival during pathogen exposure in young and middle-aged nematodes. Interestingly, *E. ulmoides* and *C. chinensis* did not show direct antibacterial effects on *P. luminescens* or *E. coli;* thus, the antimicrobial impact of these TCMs did not play a role in the observed pathogenic resistance. Other published pathogen stress resistance trials in *C. elegans* reveal possible underlying mechanisms: resistance against *Pseudomonas aeruginosa* infections has been caused by many natural or synthetic compounds that target host immune responses to attenuate infections (Pukkila-Worley et al., 2012; Kong et al., 2016). The extract from *Swietenia macrophylla* seeds, for instance, promotes the survival of *P. aeruginosa-*challenged nematodes by boosting the expression of a *C. elegans* lysozyme encoding gene (*lys-7*) (Dharmalingam et al., 2012). Also, the natural polyphenols isolated from Magnolia plant species (honokiol and magnolol) promoted a cellular immune response and slowed down *C. elegans* killing by *Staphylococcus aureus* (Choi et al., 2015). Whether a boosted immune response is also responsible for the observed effects in the current study needs to be addressed in future molecular biological experiments.

### Only *C. chinensis* Is an Overall Healthspan Enhancer

Besides stress resistance and lifespan, *C. chinensis* is also able to improve further ageing-related parameters, in fact, the pharyngeal pumping rate, locomotion and autofluorescence in older *C. elegans*.

The pharyngeal pumping rate in *C. elegans* determines the food intake and mirrors the general fitness status of the organism. *C. chinensis* exposure led to a significant increase of the pharyngeal pumping frequency of middle-aged and old *C. elegans*, whereas the treatment with *E. ulmoides* had no impact on the pumping rate. Several studies reported that an increased pharyngeal pumping is a sign of rejuvenation, since the pumping rate declines gradually with increasing age (Huang et al., 2004). Thus, the increased pumping frequency in *C. chinensis*-treated nematodes could be a sign of slower aging. This result is consistent with a recent report of Zeng et al. (2019). They investigated *Jianpi-yangwei*, a traditional Chinese medicine primarily used to treat functional decline related to aging in humans, and showed that it also slowed the decline in pharyngeal pumping during aging in *C. elegans*. In addition, polyphenols, which are suggested to be the main active TCM ingredient in our plants (Liu and Henkel, 2002), are able to increase the pumping rate as shown e.g. for blueberry polyphenols (Wilson et al., 2006). On the other hand, previous studies suggested that decreased pumping frequency is a cause of health- and lifespan enhancement in *C. elegans,* with dietary restriction as the underlying reason (Lakowski and Hekimi, 1998). However, this mode of action can be excluded for the two tested extracts since no reduction in the pumping rate or food intake was observed subsequent to the treatment.

Autofluorescent material (sometimes referred to as lipofuscin or “age pigment”) builds up over time in cells and tissues with low turnover (Terman and Brunk, 2006), and is often used as a marker of aging in *C. elegans* (Pincus and Slack, 2010). The transparent nature of *C. elegans* facilitates the examination of that autofluorescent material in living subjects (Gerstbrein et al., 2005). It was shown in *C. elegans* that the red autofluorescence, which is mainly located in the intestine, reflects the aging status of the individual in the most reliable way (Pincus et al., 2016). Our results revealed that *C. chinensis-*treated *C. elegans* showed significantly reduced autofluorescence intensities; however, the treatment with *E. ulmoides* had no impact on the accumulation of autofluorescent material. Indeed, several TCMs have been reported as important sources of active compounds that decrease the accumulation of autofluorescent material in *C. elegans,* but most are hard to compare to our work. For instance, a *Panax notoginseng* preparation astonishingly reduced the autofluorescence by roughly one half (Jin et al., 2019). However, the concentrations used were more than 25 times higher compared to the current study. Moreover, blue instead of red fluorescence was used, which is not suitable as an aging marker (Pincus et al., 2016). Also a *Jianpi-yangwei* preparation was used successfully to reduce the autofluorescence (Zeng et al., 2019), but again, the concentration was higher than in the current study, and green rather than red fluorescence was used. It remains unclear whether other TCM preparations at low concentrations exert similar activities, since the required studies have not been carried out.

Locomotion is one of the most important features that reflect the physical ability of nematodes and the aging stage (Li et al., 2019a). This study evaluated the ability of *C. chinensis* and *E. ulmoides* to upgrade the nematode’s swimming by measuring wave initiation rate, activity index, brush stroke, and body wave number. The wave initiation rate is the number of body waves per minute, which indicates the movement-speed, whereas the body wave number determines the waviness of the body at each time point. Furthermore, the activity index adds up the number of pixels that are covered by the nematode during the time spent for two strokes as an indicator for the vigorousness of bending over time. Finally, the brush stroke parameter reflects the area covered by the body in a complete stroke, which indicates the depth of the movement. Both latter parameters were normalized to the body size. These features were selected due to their strong age dependence, since the body wave number is increasing and the remaining parameters are decreasing with age (Restif et al., 2014). Interestingly*, C. chinensis* could significantly improve all measured locomotion parameters particularly in old *C. elegans*. On the other hand, *E. ulmoides* did not produce any statistically significant difference on the swimming performance of *C. elegans*. This is in line with the observations for the autofluorescence and the pharyngeal pumping, which together indicate that *E. ulmoides* is not a general anti-aging extract, but specifically acts on stress resistance. On the contrary, *C. chinensis* provoked beneficial effects in all life- and healthspan parameters and could be regarded as an overall anti-aging plant preparation.

To quantify the benefit of the extract treatments, the results were compared to quercetin. The polyphenol quercetin is a prominent and robust health- and lifespan enhancer which is studied usually in a range of 50–200 µM with overall beneficial effects in *C. elegans*. The survival after heat stress, starting between the 2^nd^ and 6^th^ day of adulthood, was increased by 8% (Zhou et al., 2018), 14% (Upadhyay et al., 2013; Büchter et al., 2015), or 20% (Saul et al., 2008), respectively, in quercetin-treated nematodes. By using comparable ages and test performances, *C. chinensis* increased the survival by 19–24% and *E. ulmoides* by 22–38%, thus, mostly exceeding the beneficial effects previously published for quercetin. Furthermore, the motility (Sugawara and Sakamoto, 2020) and pharyngeal pumping (Pietsch et al., 2011) were significantly enhanced in aged nematodes, and blue autofluorescence decreased by 28% in moderately aged nematodes (Büchter et al., 2015) after quercetin treatment. *C. chinensis* treatment resulted in similar effects, but could decrease the autofluorescence only by 4–6%. However, we measured red instead of blue autofluorescence, as suggested by Pincus et al. (2016). Thus, a direct comparison is not possible. Quercetin treatments also increase mean lifespan by 10% (Saul et al., 2008), 15% (Kampkötter et al., 2008), 18% (Pietsch et al., 2009), or 19% (Büchter et al., 2015), respectively. This is in line with our lifespan extension of 19% by 100 µM quercetin (which corresponds to about 30 μg/ml). Nematodes treated with 30 μg/ml *C. chinensis* surpass these values by more than 5%, whereas *E. ulmoides* only induced a slight life extension compared to quercetin. In total, *C. chinensis* effects seem to be comparable to quercetin, or are even somewhat stronger in terms of health- and lifespan prolongation.

Health-enhancements via TCMs only produce minor trade-off effects in reproductive traits. According to the life history theory there is a strong relationship between longevity, body size and fecundity (Fabian and Flatt, 2012). This theory suggests that an increase of body size can be balanced against the benefits of faster reproduction and the costs of lower offspring viability and lower initial fecundity, weighed against a backdrop of differential allocation of physiological and metabolic resources to each of these processes and to growth itself. (Woodruff et al., 2019). The change in both, biotic (predation pressure, resource abundance, pollinator density, etc.) and abiotic (temperature, salinity, humidity, etc.) environmental factors can have an impact on the survival, growth and fecundity of an organism (Woodruff et al., 2019). Thus, by increasing the survival through the usage of the TCM extracts, a trade-off in other life-history traits as described by Stearns (1989) and Zera and Harshman (2001) is conceivable. Therefore, we tried to exclude any side effects of *E. ulmoides* and *C. chinensis* on the growth as well as reproductive fitness of *C. elegans.* Our results revealed that *E. ulmoides* and *C. chinensis* increased the healthspan and lifespan of *C. elegans* without significantly impairing overall growth and offspring, but with a slight impact on the timing of reproduction. Some health-prolonging substances have already been found to delay or decrease reproductive output (Saul et al., 2013; Kim et al., 2014; Zhuang et al., 2014). For instance, Saul et al. (2011) and Saul et al. (2013) reported that after life- and healthspan promoting polyphenol exposures, the beneficial effects in *C. elegans* were associated with significant postponement of the initial reproduction. Moreover, other studies concluded that the lifespan and healthspan extensions caused by different substance exposures were often associated with reductions in growth of *C. elegans* (Liao et al., 2011; Saul et al., 2013). Curcumin, as an active ingredient in the herbal medicine turmeric (*Curcuma longa*), caused lifespan and healthspan extension in *C. elegans* in parallel to body size decrease (Liao et al., 2011). Furthermore, the two polyphenolic tannin components tannic acid and catechin were able to extend the life- and healthspan of *C. elegans*, but with significant reduction in the body size (Saul et al., 2013). On the other hand, several studies found healthspan prolongation as well as improved fecundity in parallel (Ryu et al., 2016; Zhang et al., 2018b). In many cases, however, enhanced survival is not linked to a trade-off in reproduction or growth traits. Scerbak and colleagues found that lifespan-extending Blueberry and fungus treatments did not influence total progeny produced per individual over reproductive lifespan (Scerbak et al., 2016). Tyrosol, a main phenol in extra virgin olive oil, increased healthspan and did not affect other fitness parameters in *C. elegans,* such as growth and reproduction. Although treated nematodes showed a slight decrease in body size at the L4 larval stage, the body size on the first and sixth adult day was not affected (Cañuelo et al., 2012).

### General Discussion and Limitations of Our Study

All the selected TCM preparations are claimed to have anti-aging activity, and most indeed show effects on some of the aging parameters that we measured in *C. elegans*. However, deeper phenotyping reveals differences in their effects. In several cases, effects are only observed at earlier ages (corresponding to adult animals). Even for the two extracts that show activity in older worms, the spectrum of effects is different. A decrease in resistance to various stresses is generally viewed as a hallmark of aging, but reversing this effect apparently does not suffice to combat also other signs of aging, like locomotor ones. This is consistent with the observation that different physiological systems (locomotor, cardiovascular, immune, etc.) may age at divergent rates in different individuals. Nonetheless, some molecular processes probably underlie aging in general, and would therefore affect all organs and tissues. Plant extracts with widespread anti-aging effects across physiological systems therefore likely affect such generic molecular aging processes, and are most attractive therapeutically.

Our study has a number of limitations. Effects in *C. elegans* do not necessarily extrapolate to other animals, even though most aging mechanisms and pathways in *C. elegans* have counterparts in mammals. Moreover, anti-aging effects of *C. chinensis* have been documented in animals, and are attested in TCM for humans (Donnapee et al., 2014). In any case, mechanistic studies will be easier in *C. elegans*. Although the precise anti-aging mechanism of the TCM extracts remains to be elucidated, differences between them have already become apparent. We only tested a single dose of the TCM plant extracts, and only a single solvent was used for extraction. It is conceivable that preparations at higher doses or prepared with other solvents could be more efficacious, but they may also prove to be more toxic. It is not clear whether the anti-aging compounds from the plant extracts are absorbed via the intestine after ingestion, or penetrate the *C. elegans* cuticle. However, in mice as well as rats, oral administration of *C. chinensis* preparations showed anti-aging effects. The plant extracts were administered to *C. elegans* from the beginning of the L4 stage; it is therefore not clear whether they would be equally effective if started at later times in life. Several of the phytochemicals documented in *C. chinensis* have anti-aging potential, but their relative contributions and potential synergies would be quite hard to study in mammals; however, this should be feasible in *C. elegans*.

## Conclusion

This study demonstrates the different effects (and possible modes of action) of the tested TCM extracts. *E. ulmoides* affected specific physiological characteristics by improving the survival probability of *C. elegans* during and after stress. On the other hand, *C. chinensis* enhanced the healthspan in a more general way by improving the physical fitness, the stress resistance and the “autofluorescence” aging biomarker. In addition, *C. chinensis* exposure led to a stronger life-prolonging effect under standard laboratory conditions compared to *E. ulmoides,* and the upregulation of *hsp-16.1* was only observed after *C. chinensis* treatment. Furthermore, extracts from *L. lucidum, P. cocos, A. membranaceus, S. chinensis*, and *A. bidentata* were beneficial for *C. elegans* in stress conditions at younger age. The analysis of additional endpoints and/or concentrations could further uncover their potential and their relation to a hormesis effect. Collectively, our results suggest that TCM extracts have a promising capability to modulate healthspan in a beneficial way, with *C. chinensis* having the greatest potential to be an effective antiaging treatment out of the seven extracts tested.

## Data Availability Statement

The original contributions presented in the study are included in the article/Supplementary Material, further inquiries can be directed to the corresponding author.

## Author Contributions

Conceptualization, SS, CS-L, WL, and NS; methodology, SS and NS; validation, NS and CS-L; formal analysis, SS; investigation, SS; resources, KS; data curation, SS and NS; writing—original draft preparation, SS; writing—review and editing, CS-L, WL, KS, and NS; visualization, SS; supervision, NS and CS-L; project administration, WL, CS-L, and NS; funding acquisition, WL, CS-L, and NS All authors have read and agreed to the published version of the manuscript.

## Funding

This project has received funding from the European Union’s Horizon 2020 research and innovation program under Grant agreement No 633589 (Aging with Elegans). This publication reflects only the authors’ views and the Commission is not responsible for any use that may be made of the information it contains. In addition, a financial support from the Yousef Jameel Academic Program at the Humboldt-University of Berlin, Germany is gratefully acknowledged. Finally, we acknowledge support by the German Research Foundation (DFG) and the Open Access Publication Fund of Humboldt-Universität zu Berlin.

## Conflict of Interest

Author KS was employed by the company AnalytiCon Discovery GmbH, Germany.

The remaining authors declare that the research was conducted in the absence of any commercial or financial relationships that could be construed as a potential conflict of interest.
