# Supplementary material for: Enhanced Healthspan in Caenorhabditis elegans Treated With Extracts From the Traditional Chinese Medicine Plants Cuscuta chinensis Lam. and Eucommia ulmoides Oliv
Source: Front Pharmacol. 2021 Feb 2;12:604435. doi: 10.3389/fphar.2021.604435 (PMC7901915; doi:10.3389/fphar.2021.604435)
Supplement: Supplementary file 1 [file datasheet1.pdf]

Table S1: HPLC-MS method for the analysis of plant extracts

**HPLC System**	PE Series 200		
**MS System**	Applied Biosystems API 150		
**Data System**	Analyst 1.3		
**Stationary Phase**	Merck Select B 250x4 mm, 5 □m		
**Flow Rate**	1 mL/min		
**Detection**	(+/(-)-ESI, Fast-Switching-Mode<br>ELSD (Sedex 75)<br>UV (DAD 200 – 400 nm)		
**Sample concentration**	10 mg/mL in DMSO		
**Injection Volume**	30 µL		
**Mobile Phase:**	A: 5 mM ammoniumformate and 0.1 % formic acid<br>B: acetonitrile/methanol = 1:1, 5 mM ammoniumformate and 0.1 % formic acid (pH 3)		
**Gradient**	Time [min]	% A	% B
	00.0	85	15
	30.0	0	100
	35.0	0	100

Table S2: MS settings

Source Type:	Turbo Spray
Source Temperature (at setpoint):	350.0 °C

(+)/(-)-ESI switching mode:

<u>Experiment 1:</u>

Scan Type:	Q1 MS (Q1)
Polarity:	Positive
Scan Mode:	Profile
Resolution Q1:	UNIT
Settling Time:	700 ms
Start (amu):	150
Stop (amu):	1500
NEB:	6
CUR:	10
IS:	4200
TEM:	350
DP	40
FP	200
EP	10

<u>Experiment 2:</u>

Scan Type:	Q1 MS (Q1)
Polarity:	Negative
Scan Mode:	Profile
Resolution Q1:	UNIT
Settling Time:	700 ms
Start (amu):	150
Stop (amu):	1500
NEB:	6
CUR:	10
IS:	-4500
TEM:	350
DP	-70
FP	-220
EP	-10

DAD settings: 210 to 400 nm, width 2 nm

Table S3: Main compounds identified in the *C. chinensis* extract

t_R [min]	area %	MW	compounds
<5			sugars
11,80	1,5	596	quercetin glycoside
12,73	2,8	464	quercetin glycoside
13,97	0,6	448	astragalin
19,50	1,0	798	Cus 3 or isomer
20,36	0,8	798	Cus 3 or isomer
21,34	1,3	840	Cus 1 or isomer
22,19	3,5	840	Cus 1 or isomer
23,79	0,6	868	Cus 2 or isomer
>30			fatty acids, glycolipids, phospholipids

Glycoretins:

Cus 1: R = CH_3

Cus 2: R = $CH_2CH_2CH_3$

Cus 3: Deacetyl-Cus 1

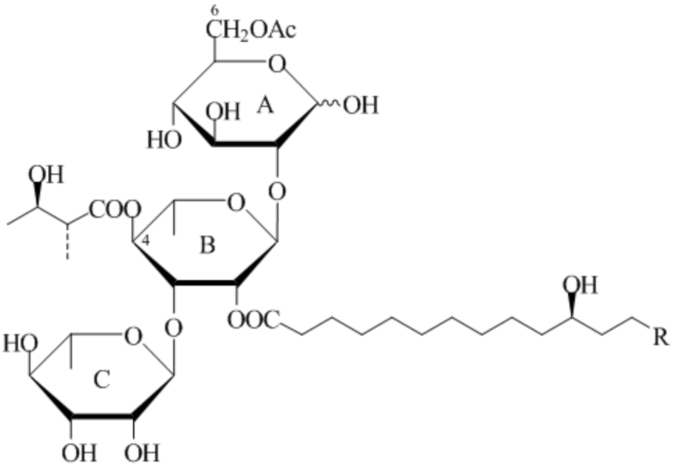

Table S4: Primer for the qPCR assay

Primer name	Sequence [5‘ to 3‘]	Annealing temperature [°C]	Product length [bp]	Efficiency
act-1 fw	TCCAAGAGAGGTATCCTTAC	56	169	98,3
act-1 rv	CGGTTAGCCTTTGGATTGAG			
cdc-42 fw	ATTACGCCGTCACAGTAATG	56	248	93,7
cdc-42 rv	ATCCCTGAGATCGACTTGAG			
hsp-16.1 fw	CTATTTCCGTCCAGCTCAAC	54.8	410	95.2
hsp-16.1 rv	TTTGTTCAACGGGCGCTTGC			
hsp-16.2 fw	ACGCCAATTTGCTCCAGTCT	55	347	99.7
hsp-16.2 rv	TCTCTTCGACGATTGCCTGT			
hsp-70 fw	AGCCGGTTGAAAAGGCACT	55	338	97.4
hsp-70 rv	TGTTTTGGAAGCTTTGGCAGG			
daf-21 fw	ATTCGCTACCAGGCACTCAC	55.9	240	101.7
daf-21 rv	GAATCCGACTCCGAACTGAC			
hsp-12-6 fw	TGGAGTTGTCAATGTCCTCG	53.6	235	100.3
hsp-12-6 rv	TCCATGTGAATCCAAGTTGCTC			

Table S5: qPCR thermal cycling protocol

Step	Minutes
Step 1: 95°C	02:00
Step 2: 95°C	00:10 } 35 loops
Step 3: Ta°C	00:20 } 35 loops
Step 4: 72°C	00:30 } 35 loops
Step 5: 95°C	01:00
Step 6: Melt curve: Ta°C – 95°C, steps: 0.5 °C	00:10 per temperature step

Ta = Annealing temperature

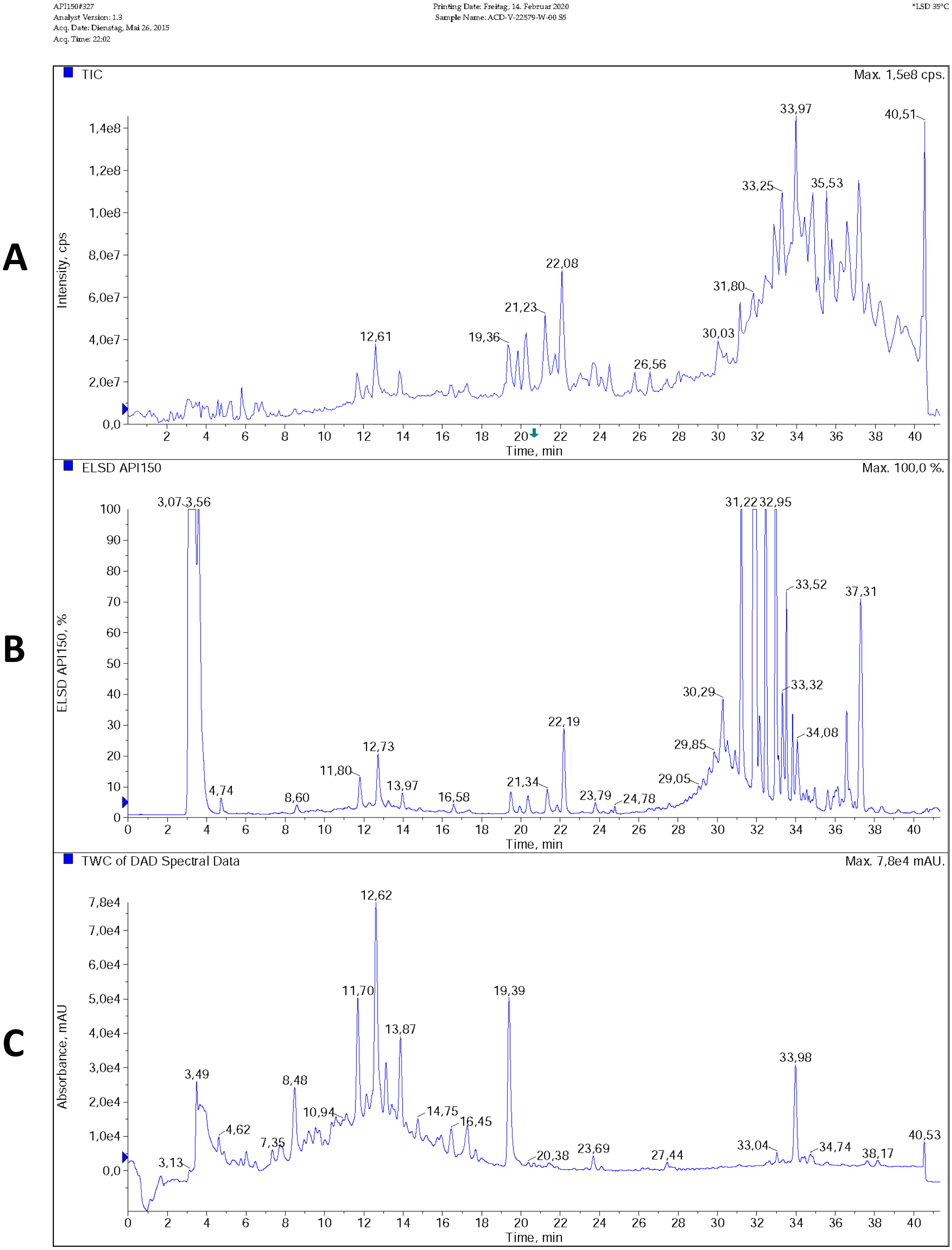

Figure S1: HPLC-MS analysis of the *C. Chinensis* extract (ACD-V-22579-W-00)

(A) The total-ion chromatogram (TIC), **(B)** the evaporative light scattering detector (ELSD) chromatogram and **(C)** the total wavelength chromatogram (TWC) using a diode array detector (DAD) are shown.

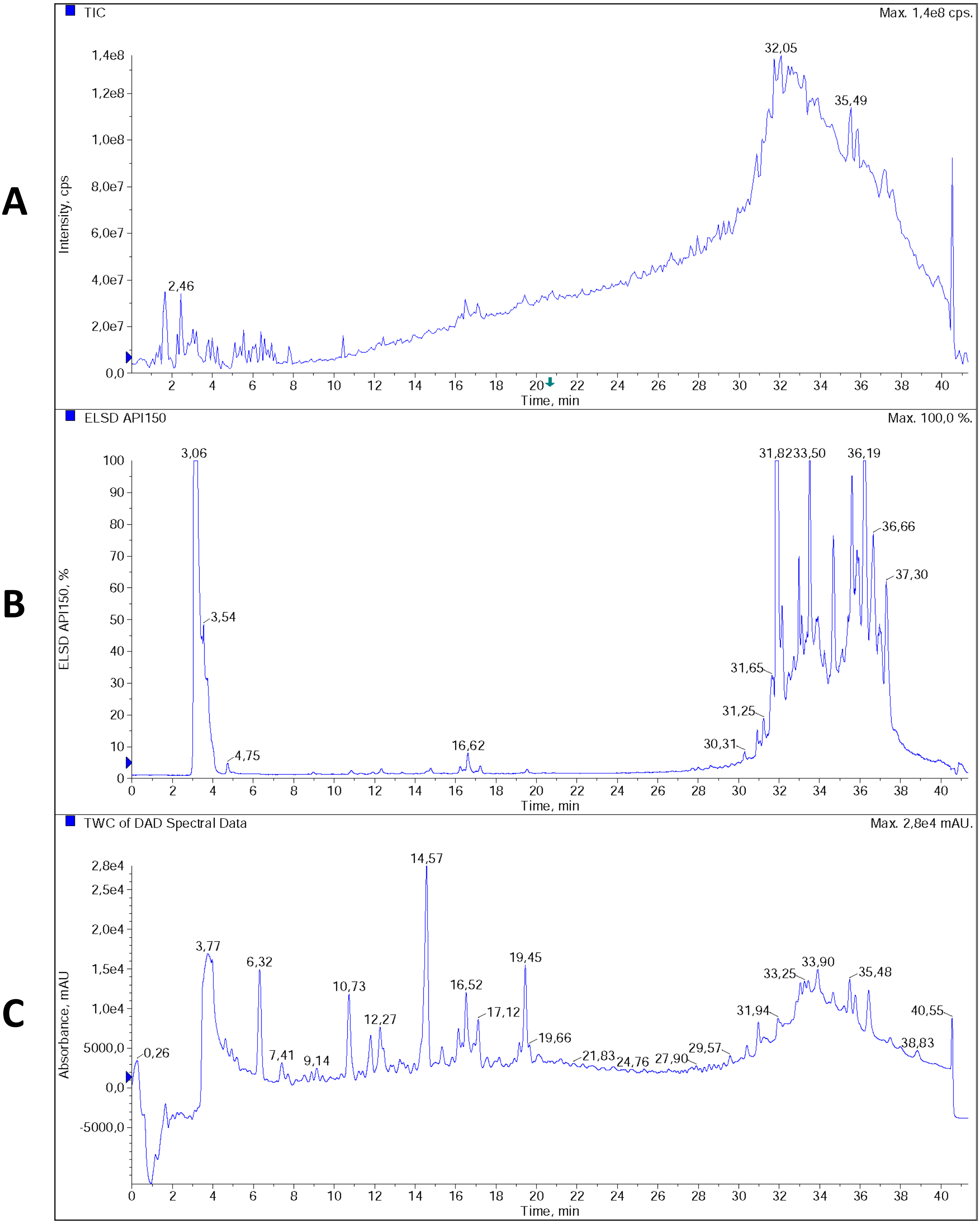

Figure S2: HPLC-MS analysis of the *E. Ulmoides* extract (ACD-V-22582-W-00)
(A) The total-ion chromatogram (TIC), **(B)** the evaporative light scattering detector (ELSD) chromatogram and **(C)** the total wavelength chromatogram (TWC) using a diode array detector (DAD) are shown.

API150#327
Analyst Version: 1.3
Acq. Date: Dienstag, Mai 26, 2015
Acq. Time: 23:28

Printing Date: Freitag, 14. Februar 2020
Sample Name: ACD-V-22580-W-00 S5

*LSD 35°C

A

B

C

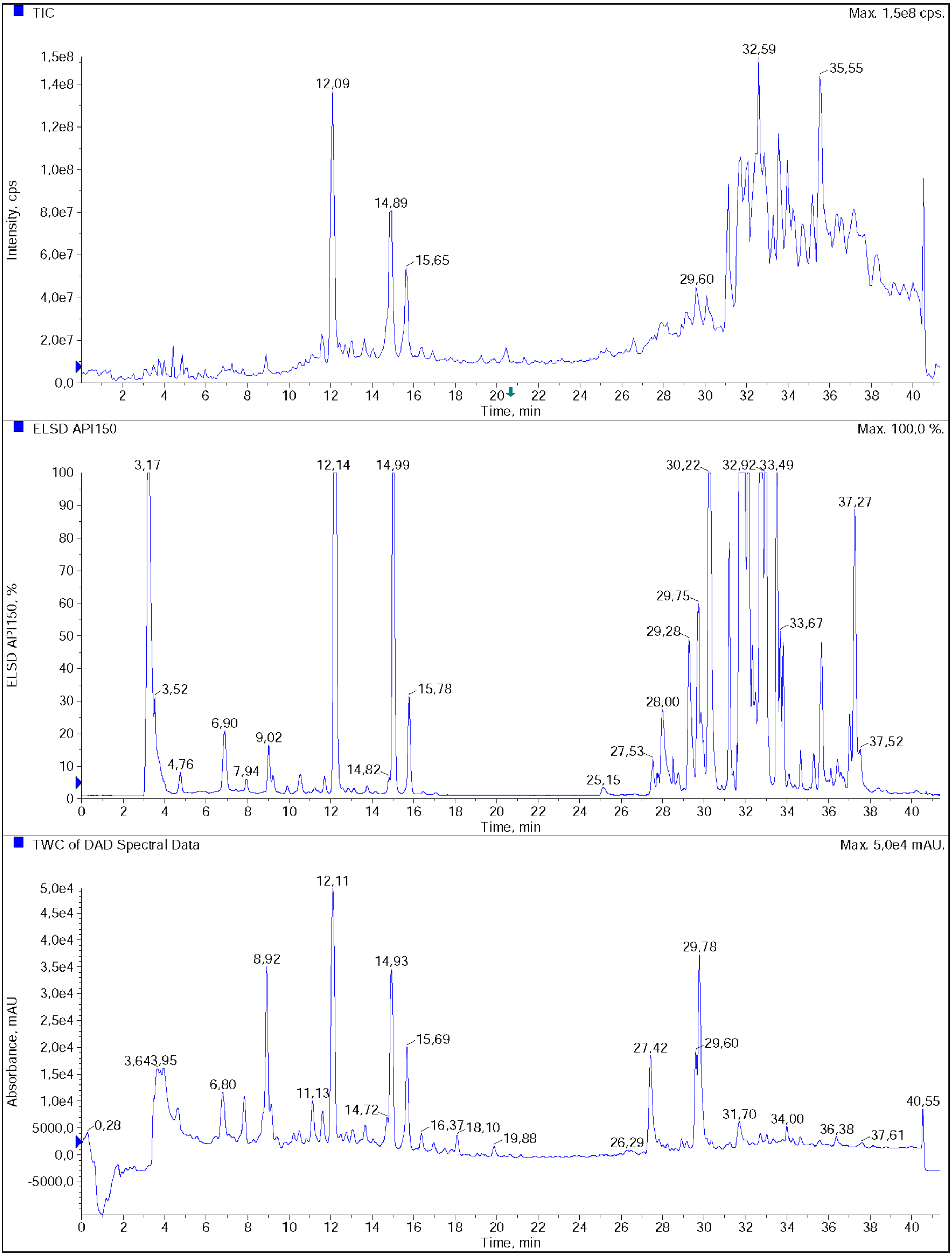

Figure S3: HPLC-MS analysis of the *L. Lucidum* extract (ACD-V-22580-W-00)

(A) The total-ion chromatogram (TIC), **(B)** the evaporative light scattering detector (ELSD) chromatogram and **(C)** the total wavelength chromatogram (TWC) using a diode array detector (DAD) are shown.

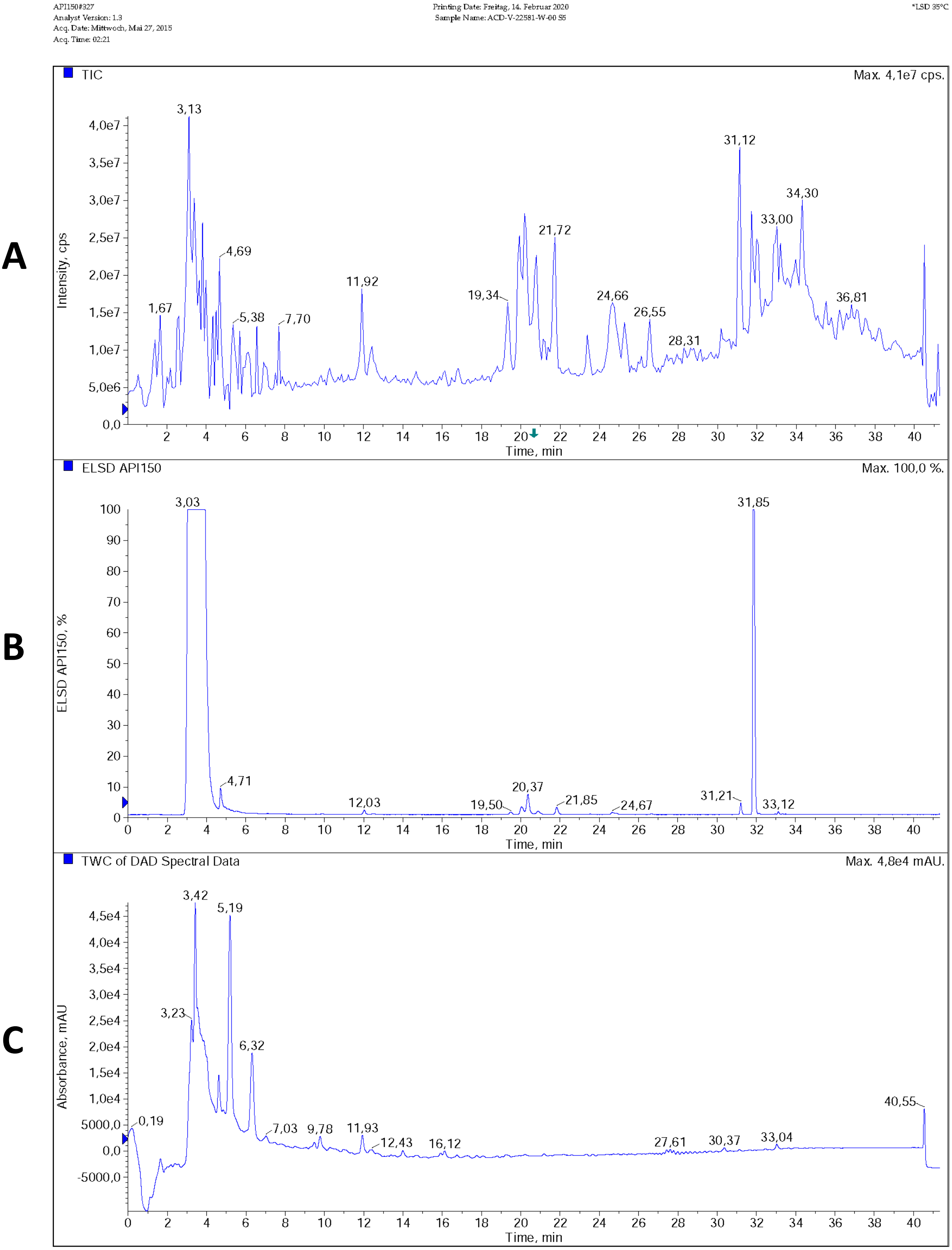

Figure S4: HPLC-MS analysis of the *A. Bidentata* extract (ACD-V-22581-W-00)

(A) The total-ion chromatogram (TIC), **(B)** the evaporative light scattering detector (ELSD) chromatogram and **(C)** the total wavelength chromatogram (TWC) using a diode array detector (DAD) are shown.

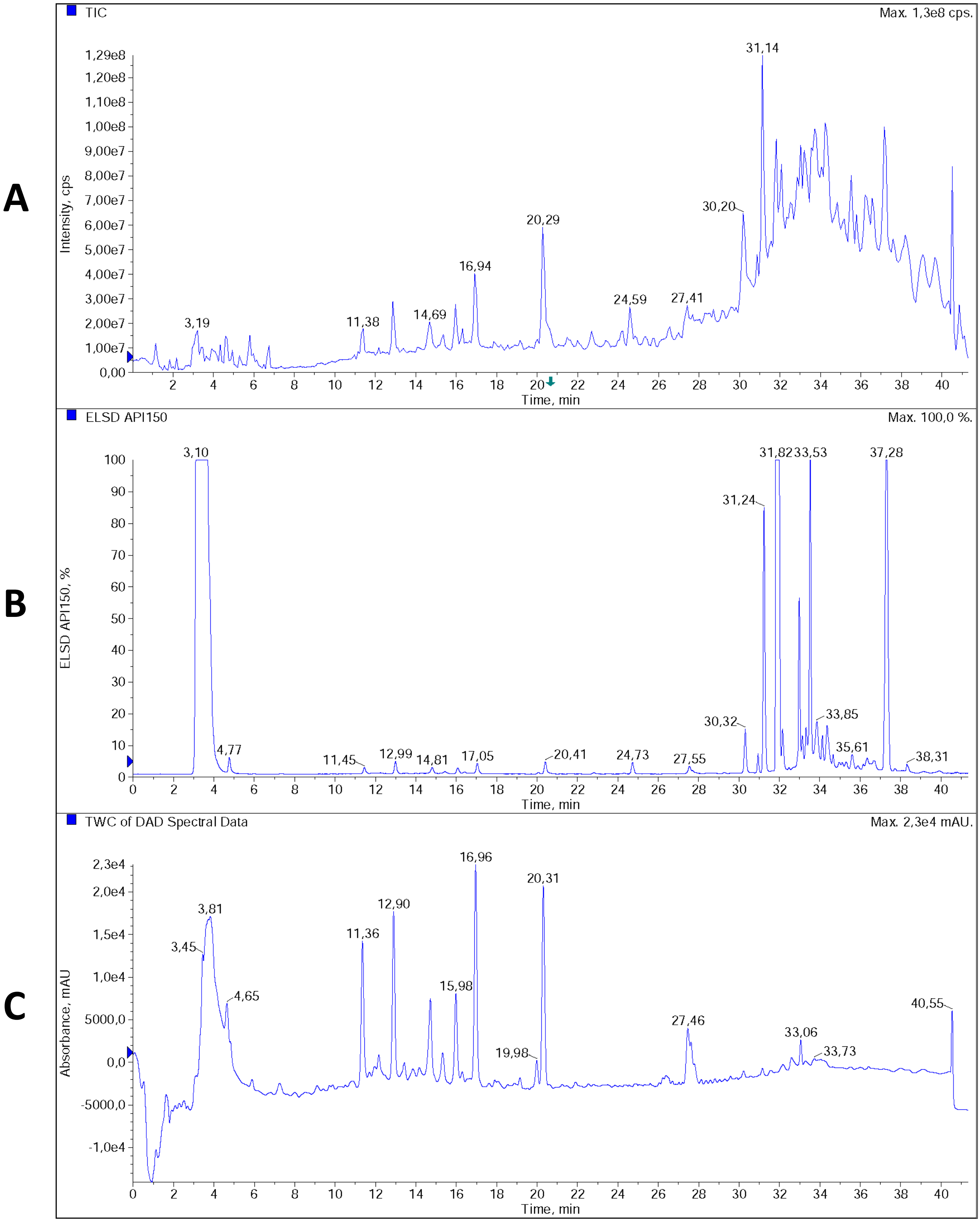

Figure S5: HPLC-MS analysis of the *A. Membranaceus* extract (ACD-V-22577-W-00)
(A) The total-ion chromatogram (TIC), **(B)** the evaporative light scattering detector (ELSD) chromatogram and **(C)** the total wavelength chromatogram (TWC) using a diode array detector (DAD) are shown.

API150#327
Analyst Version: 1.3
Acq. Date: Mittwoch, Mai 27, 2015
Acq. Time: 12:25

Printing Date: Freitag, 14. Februar 2020
Sample Name: ACD-V-22587-W-00 S5

*LSD 35°C

A

B

C

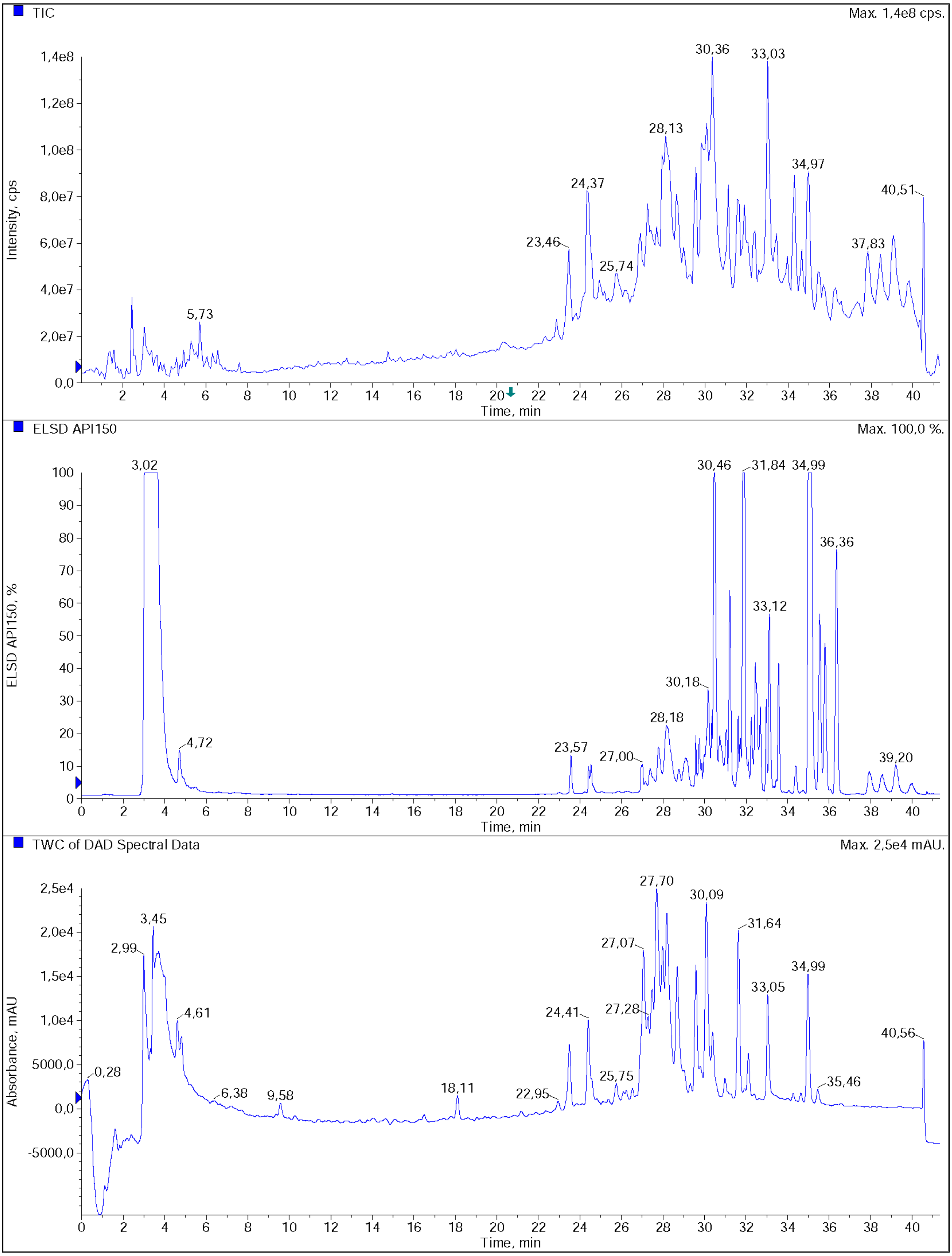

Figure S6: HPLC-MS analysis of the *P. Cocos* extract (ACD-V-22587-W-00)

(A) The total-ion chromatogram (TIC), **(B)** the evaporative light scattering detector (ELSD) chromatogram and **(C)** the total wavelength chromatogram (TWC) using a diode array detector (DAD) are shown.

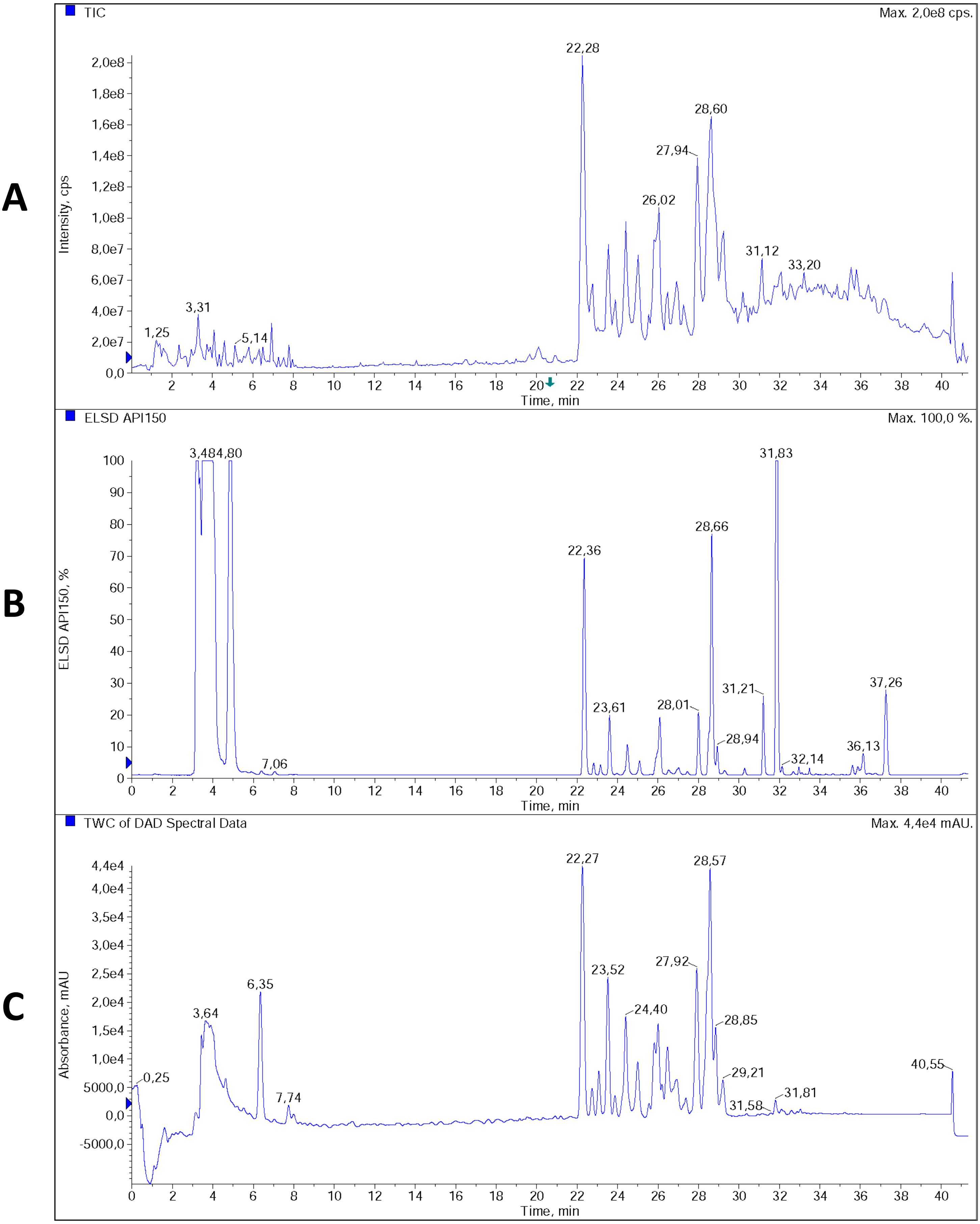

Figure S7: HPLC-MS analysis of the *S. Chinensis* extract (ACD-V-22584-W-00)

(A) The total-ion chromatogram (TIC), **(B)** the evaporative light scattering detector (ELSD) chromatogram and **(C)** the total wavelength chromatogram (TWC) using a diode array detector (DAD) are shown.
